# Space oddity: musical syntax is mapped onto visual space

**DOI:** 10.1038/s41598-021-01393-1

**Published:** 2021-11-16

**Authors:** Neta B. Maimon, Dominique Lamy, Zohar Eitan

**Affiliations:** 1 The School of Psychological Sciences, Tel Aviv University, 69978 Tel Aviv, Israel; 2 The Sagol School of Neuroscience, Tel Aviv University, 69978 Tel Aviv, Israel; 3 The Buchmann Mehta School of Music, Tel Aviv University, 69978 Tel Aviv, Israel

**Keywords:** Human behaviour, Perception

## Abstract

Increasing evidence has uncovered associations between the cognition of abstract schemas and spatial perception. Here we examine such associations for Western musical syntax, *tonality.* Spatial metaphors are ubiquitous when describing tonality: stable, closural tones are considered to be spatially central and, as gravitational foci, spatially lower. We investigated whether listeners, musicians and nonmusicians, indeed associate tonal relationships with visuospatial dimensions, including spatial height, centrality, laterality, and size, implicitly or explicitly, and whether such mappings are consistent with established metaphors. In the explicit paradigm, participants heard a tonality-establishing prime followed by a probe tone and coupled each probe with a subjectively appropriate location (Exp.1) or size (Exp.4). The implicit paradigm used a version of the Implicit Association Test to examine associations of tonal stability with vertical position (Exp.2), lateral position (Exp3) and size (Exp.5). Tonal stability was indeed associated with perceived physical space: the spatial distances between the locations associated with different scale-degrees significantly correlated with the tonal stability differences between these scale-degrees. However, inconsistently with musical discourse, stable tones were associated with leftward (instead of central) and higher (instead of lower) spatial positions. We speculate that these mappings are influenced by emotion, embodying the “good is up” metaphor, and by the spatial structure of music keyboards. Taken together, the results demonstrate a new type of cross-modal correspondence and a hitherto under-researched connotative function of musical structure. Importantly, the results suggest that the spatial mappings of an abstract domain may be independent of the spatial metaphors used to describe that domain.

## Introduction

Theoretical models and empirical studies in cognitive science and linguistics have long suggested that our cognition of abstract concepts and schemata is grounded in the perceived structure of concrete physical domains, particularly aspects of space. Lakoff and Johnson’s Conceptual Metaphor Theory (CMT^1,2^) famously proposed that fundamental concepts in apparently abstract domains, such as emotion, morality, and social hierarchy are systematically organized in terms of spatial metaphors. For instance, vertical orientation (up/down) is used to signify mood (e.g., cheer up, I am feeling down), state of consciousness (wake up), moral virtue (upright) or status (a high position).

Importantly, such mappings may shape not only how we speak about abstract domains (i.e., the relevant metaphorical vocabulary) but also cognitive processes not explicitly involving language. Though CMT’s notion of conceptual metaphor is not universally accepted^3^, the role of spatial mappings, as well as of other basic perceptual dimensions, such as brightness, in processing abstract concepts and schemata has been supported by ample behavioral research. Consider, for instance, the correspondence between spatial elevation and valence. A word’s valence affects spatio-visual attention, such that positive words shift attention upwards, and negative words—downwards^4^. Learning of novel positive and negative words also is enhanced when these are encoded at high and low spatial locations, respectively^5,6^. Likewise, moving objects up or down enhances recall of positive and negative episodic memories, respectively^7–9^. Finally, valence biases recall of spatial location: positive images are recalled at higher locations, and negative images—at lower locations relative to where they actually appeared^10^. Recent imaging studies support such behavioral findings, suggesting that “the same neuronal coding schemes characterizing navigation in the physical space … underlie navigation of abstract semantic spaces”^11^—thus supplying a neurophysiological basis to the notion that our abstract thinking may be modeled upon spatial relationships.

### Tonality as musical syntax

The present behavioral study extends previous research mapping abstract concepts onto perceived space to a new domain: Western musical tonality. Since the 17th century, the structure of most Western music has been strongly governed by *tonality*—a system of pitch relationships, explicitly codified by music theorists and implicitly processed by listeners. Tonality is a system of interrelationships among tones and chords within major or minor keys, all relating to a single tone and chord, the tonic. The tonic note (scale degree 1) and, to a lesser extent, the other members of the tonic chord (scale degrees 3 and 5) are considered most stable and closural and elicit a relatively weak tendency to proceed to another tone. The other scale degrees (2, 4, 6 and 7) are less stable, and imply continuation to their more stable neighbors, 1, 3, or 5; the remaining five chromatic (“out of key”) tones are the least stable, and strongly imply continuation to adjacent diatonic (within-key) notes (for a brief primer on tonality, see supplementary materials A).

Tonality is, in important ways, a musical syntax: a set of rules and practices, implicitly understood by listeners, which governs closure and continuity, stability and tension, in melodic and harmonic sequences^12,13^. It thus serves to shape listeners’ melodic and harmonic expectancies and governs their evaluation of how well a given tone or chord fits a musical context, as well as their evaluation of the musical tension such tone evokes (for reviews of tonal cognition research see^14–16^). Accordingly, tonality plays an important role in establishing music’s emotional connotations. (e.g.^17–19^).

### Spatial metaphors for tonality

Similarly to metaphorical mappings of other abstract domains, musicians habitually conceptualize tonal relationships via spatial and spatio-kinetic metaphors (see^20,21^ for reviews). In fact, one can hardly refer to tonal relationships without recourse to terms derived from the spatial domain. For instance, expressions such as “tonal center” (for the tonic note or chord) “leading tone” (for the scale degree resolving to the tonic), or “cadence” (a closural harmonic progression, from the Latin cadere, to fall) are ubiquitous in musicians’ discourse of tonal structure. Metaphors involving space and motion have also shaped tonal theory historically, since its inception. Tonal relationships were discussed and modelled in terms of forces operating in physical space such as gravity or magnetism, or via basic spatial relations such as center/periphery or top/bottom (for a historical review, see^22^).

Two prominent (and often linked) metaphors for tonal stability, both historically and in musicians’ discourse, are the *center/periphery* mapping, where stable scale degrees, primarily the tonic (”tonal center”) are more central than unstable degrees, and harmonic progressions move away from or toward the central tonic (e.g.^23,24^); and the *gravity* metaphor, in which the tonic and other relatively stable tones or chords generate a “gravitational” pull on nearby unstable tones or chords (e.g.^25–27^. Note that the gravity metaphor implies that stable tones are localized *below* the unstable tones pulled toward them. This association of tonal stability with spatially lower position is also supported by basic musical expressions such as “cadence” (see above), and reflected in Western musical practice, which indeed commonly uses pitch “descent” in association with closure and repose^27^.

**Figure 1:**
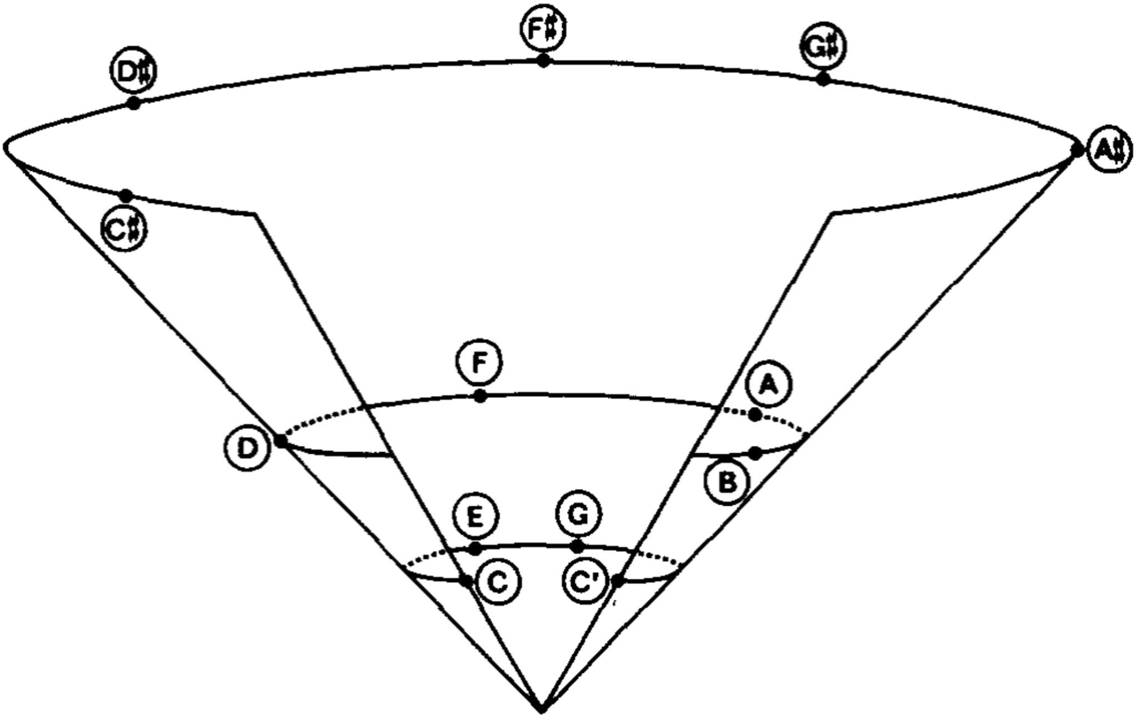
Geometrical representation of perceived similarity between musical pitches within the octave in a tonal context. C major key serves as a reference, such that C represents the tonic (scale degree 1), and C′ the pitch one octave above C. Taken from Krumhansl^28^, Fig. 3.

Interestingly, the centricity and gravity metaphors are also reflected in the way models of tonal cognition depict perceived tonal relationships geometrically. For instance, Krumhansl (^28^, see also^29^ for a comparable mapping of tonal space), modelling the stability hierarchy and perceived similarities among scale degrees (Fig. 1), position stable scale degrees at the spatial center and bottom of a three-dimensional space. Obviously, such renditions of “tonal pitch space”^29^ were not meant to be interpreted literally, in perceptual terms: Fig. 1, for example, does not suggest that the tone C# is actually perceived as located above and to the left of F. However, it is precisely such literal sense of perceived tonal space that the present study explores. We examine whether tonal relationships are actually associated by listeners (both musicians and non-musicians) with physical space, visually perceived: for instance, are more stable scale degrees perceived as situated above or below, to the right or to the left, of unstable scale degrees?

Finding that tonal syntax may engender systematic associations with perceptual space would be intriguing for two main reasons. First, while previous studies have already shown that abstract conceptual structures are modeled upon our experience of concrete physical dimensions^1–11^, music tonality differs in an important way from conceptual domains previously examined in this context, such as emotional valence or social hierarchy: For most people, the notion of tonality, and the concepts associated with it, are entirely opaque. While people with no musical training (presumably, the vast majority of the population) may implicitly respond to tonal relationships in ways consistent with tonal theory^14–16^, they have no explicit notion of tonal relationships; they are not only ignorant of the rules specified by tonal theorists but cannot explicitly identify any of tonality’s constituents (e.g., scale degrees), and lack any appropriate verbal (or otherwise symbolic) concepts to denote them.

Second, finding that tonality is associated with perceived physical space also constitutes evidence for a new type of auditory-visual cross-modal associations. While cross-modal associations have been widely investigated and corroborated between basic auditory parameters, such as pitch and loudness, and visual dimensions, such as spatial location (for research reviews see^30–32^), any such associations involving tonal syntax have not. Such associations would suggest a different, novel domain of cross-modal research, exploring correspondences between syntactic structure and perceptual dimensions. A recent study by our group^33^ was the first to demonstrate such associations, by showing that tonal hierarchy maps onto a concrete aspect of the perceived physical world, namely, brightness. We showed that listeners systematically associate more stable, closural tonal progressions with brighter visual stimuli, both explicitly and implicitly.

The present study uses the same experimental paradigms to explore the spatial associations of tonal syntax, and examines correspondences between perceived two-dimensional space and the stability hierarchy of melodic scale-degrees. We tested several hypotheses. We asked whether, generally, tonal stability consistently associates with perceived space; in particular, whether perceived physical distances of scale degrees from the tonic would negatively correlate with their level of tonal stability (Hypothesis #1). We also investigated whether correspondences between tonal stability and perceived space more specifically reflect the mappings suggested by musicians’ center-periphery and gravity metaphors. According to this musical discourse, stable tones should be perceived as located lower in physical space (Hypothesis #2) and/or in more central positions (Hypothesis #3), and perceived as being larger (Hypothesis #4). Since only musically-trained participants should be aware of these metaphors (and of tonal concepts generally) we expected these relationships to be stronger for musicians, relative to non-musicians, particularly when experimental paradigms tapping explicit judgments are used.

**Figure 2:**
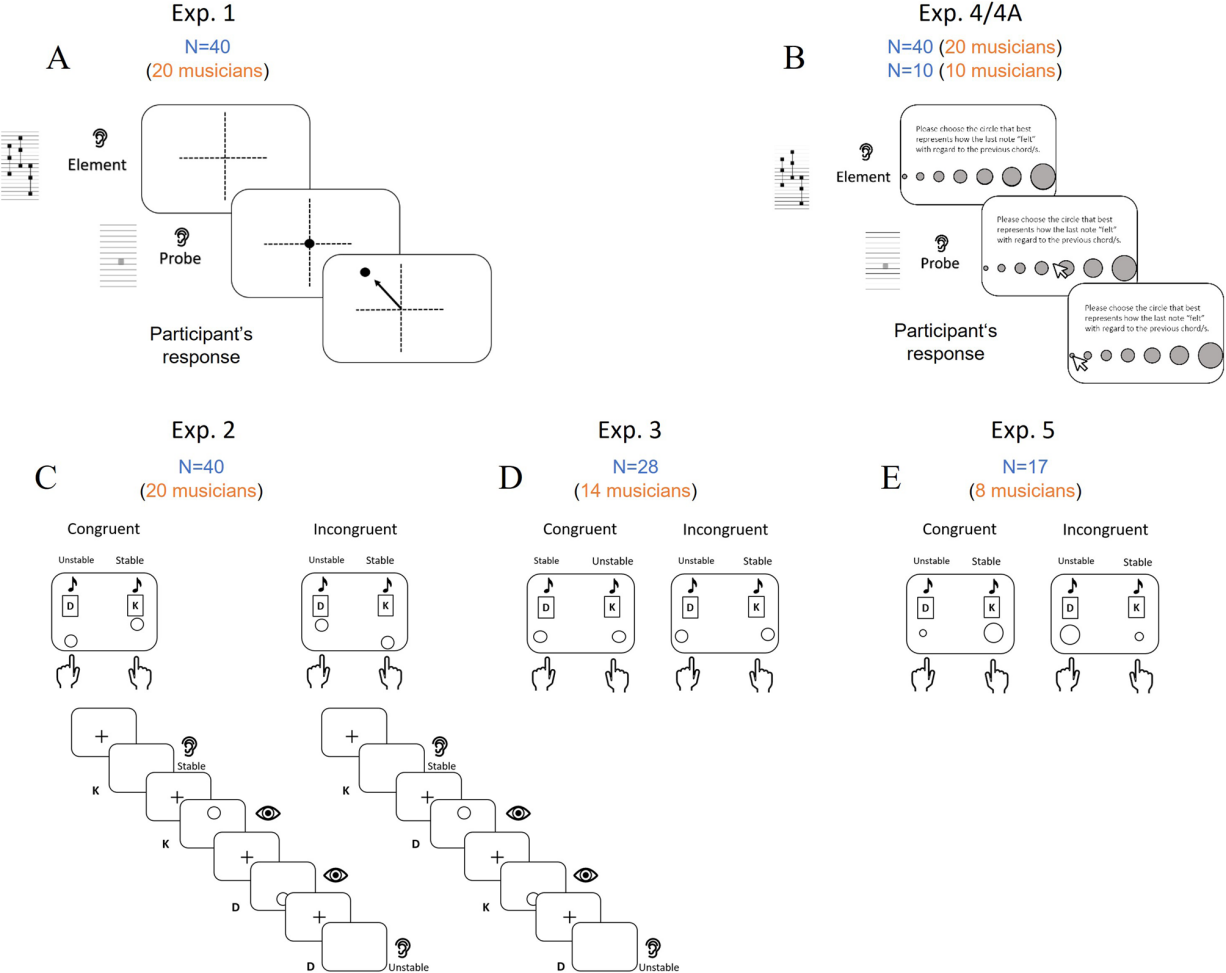
Graphic representation of the experimental designs in the present study. (**A**) Trial sequence in Experiment 1, probe tone paradigm^34^ measuring tones’ perceived location. (**B**) Trial sequence in Experiments 4 and 4A, probe tone paradigm measuring tones’ size matchings. (**C**) Block opening screen and trial sequence of congruent (left) and incongruent (right) blocks in Experiment 2, cross modal IAT^35^ measuring compatibility between tonal stability and vertical location. (**D**) Block opening screens of congruent (left) and incongruent (right) blocks in Experiment 3, cross modal IAT measuring compatibility between tonal.

We investigated these hypotheses in six behavioral experiments, using both explicit and implicit measures. Figure 2 shows the experimental design. The explicit paradigm (Experiments 1, 4, 4a) applied a version of the probe-tone method^34^. On each trial, participants heard a tonality-establishing prime (a chord sequence), followed by a probe tone (one of the 12 chromatic tones). In Experiment 1, we examined the association of tonal stability with distance from the tonic (Hypothesis 1), vertical position (Hypothesis 2), lateral position, and centrality (Hypothesis 3). Participants were asked to associate each probe tone with the location they felt was most appropriate on a two-dimensional grid. In Experiments 4 and 4a, we examined the association of tonal stability with physical size (Hypothesis 4). Participants matched each probe with one of 7 circles differing in size.

The implicit paradigm was a variant of Parise and Spence’s^35^ cross-modal version of the Implicit Association Test (IAT). We examined implicit associations of tonal stability with vertical position (Experiment 2), lateral position (Experiment 3) and object size (Experiment 5). In these experiments, a tone (either tonally stable or unstable) and a visual image (e.g., a circle either high or low in space) were assigned to the same key on the computer keyboard in hypothetically congruent (e.g., stable/low, unstable/high) or incongruent (e.g., stable/high, unstable/low) combinations. Participants were asked to press the assigned key as fast as possible in response to each tone or image. Finding that performance is better for congruent than for incongruent combinations would provide evidence that tonal stability is associated with the spatial dimensions examined. To explore the role of conceptual musical knowledge in these associations, all experiments tested both musically trained and musically untrained participants.

It should be noted that the implicit and automatic nature of responses in the IAT paradigm has been called into question, based on claims that in most IATs, the research objectives are not sufficiently concealed (e.g.^36^), that it is possible to influence IAT’s scores with deliberate thinking (e.g., ^37^) and even to fake results upon request^38^. However, these criticisms apply mainly to IATs that focus on easily-recognized attributes, in particular, attributed laden with social significance (such as ethnicity or gender). They are less likely to apply to the perceptual associations examined here, especially considering that 50% of our participants (non-musicians) had no conceptual knowledge about tonal stability, the critical auditory feature in our research.

## Results

### Localization in two-dimensional space

#### Experiment 1

Using a variant of the probe-tone method^34^, we had 40 participants (20 musicians) select the subjectively appropriate location for each of the 12 chromatic scale degrees on a two-dimensional space.

**Figure 3:**
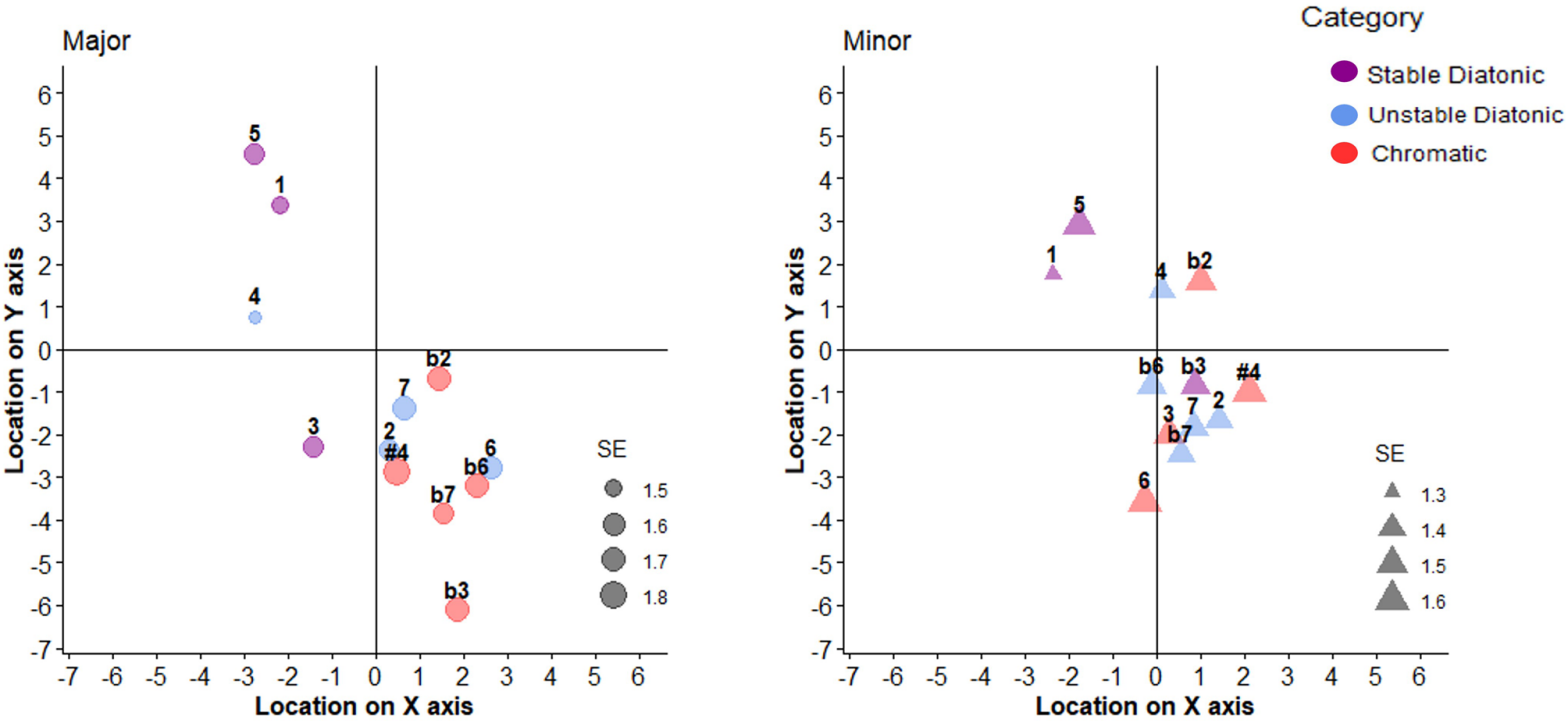
Mean localizations of each Major (left panel, circles) and Minor (right panel, triangles) scale degrees in semitones, in Experiment 1, as a function of tonal stability category (stable diatonic = purple, unstable diatonic = blue, and chromatic = red) and standard errors (relative size). Scale degrees are normalized into C Major and C minor (i.e., 1 = the tonic scale degree), # symbols represent raised degrees (e.g., #4 = = the triton), and b symbols represent lowered degrees (e.g., b2 = = lowered second). On both axes, the means never exceeded the [− 7, + 7] window presented here (the full scale ranged between – 50 and + 50).

**Figure 4:**
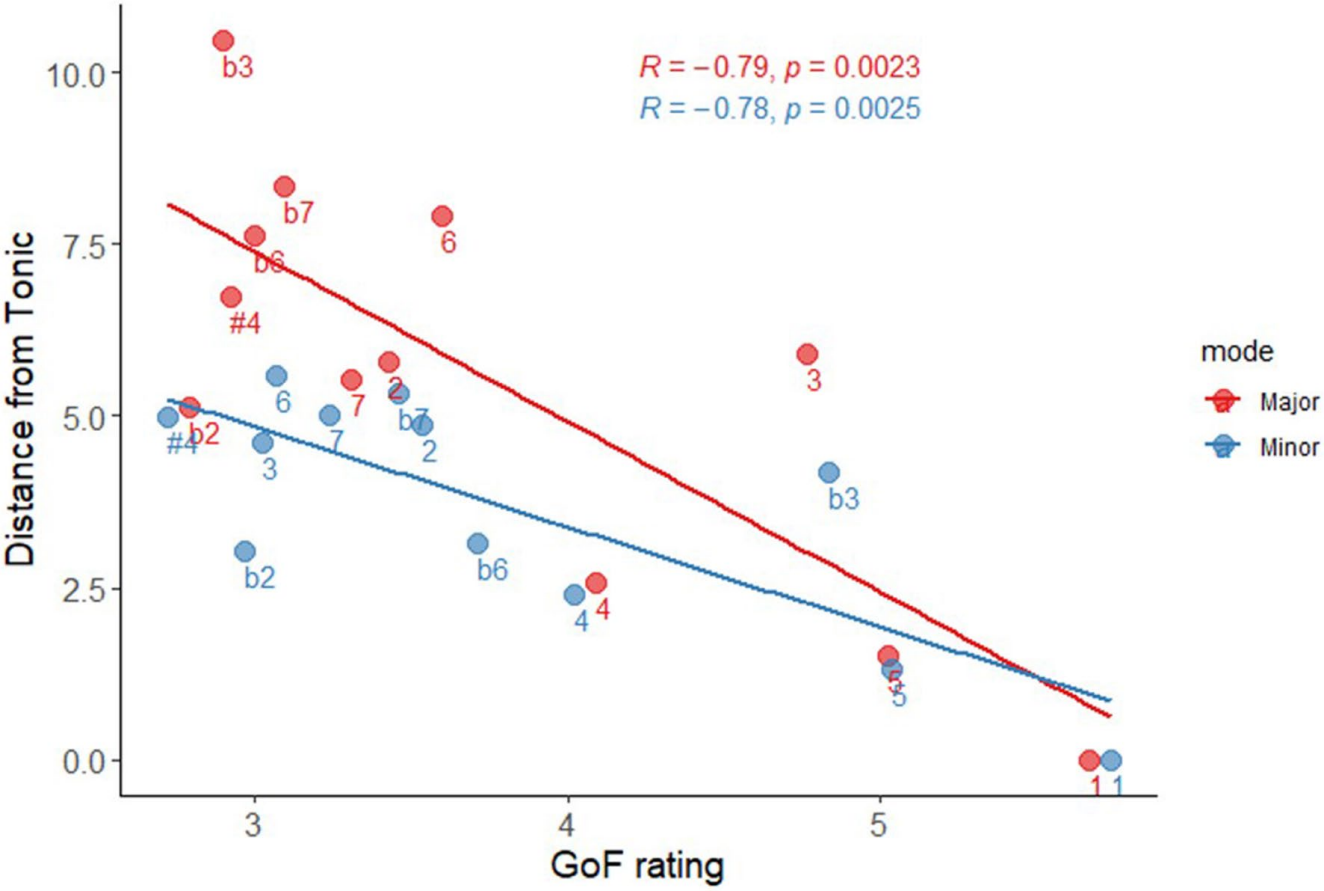
Mean distance from the tonic of each scale degree in Major (red) and Minor (blue), as a function of GoF rating (independently obtained in ref.^33^), in Experiment 1. Scale degrees are normalized into C Major and C minor (i.e., 1 = the tonic scale degree), # symbols represent raised degrees (e.g., #4 = the triton), and b symbols represent lowered degrees (e.g., b2 = = lowered second).

Mean localizations for each scale degree are presented in Fig. 3. We first addressed Hypothesis #1 by examining whether the perceived spatial distances between scale-degrees correlated with their tonal stability. To do so, we first calculated the distance between the locations associated with each scale degree (12 in major and 12 in minor) and the location associated with the tonic (the maximally stable degree), in the present experiment. We then calculated the correlation between these distances and mean goodness-of-fit (GoF) ratings, independently obtained in Maimon et al. (ref.^33^, Exp. 1), which used identical auditory stimuli. These ratings, which are similar to those obtained in “classic” GoF experiments (e.g.^34^), closely correspond with the stability hierarchy described by tonal theories. The obtained Pearson correlations validated our hypothesis: scale degrees with lower GOF ratings (i.e., which were rated as less fitting to a tonal context), were also located further away from the tonic: r = − 0.79, *p* = 0.002, and r = − 0.78, *p* = 0.0025 for major and minor modes, respectively (see Fig. 4).

Next, to explore the centrality hypothesis (Hypothesis #3), we calculated the radial distance of each scale degree from the axes’ origin (0,0 point) and averaged it separately for each of the three tonal stability categories (i.e., stable diatonic, unstable diatonic and chromatic; see materials and methods for further details). Unstable diatonic tones were located closest to the axes’ origin (x axis M = 0.398, SD = 8.68, y axis M = − 1.24, SD = 10.41, radial distance = 1.31), followed by stable diatonic tones (x axis M = − 1.595, SD = 8.76, y axis M = 1.57, SD = 10.25, radial distance =2.24), with the chromatic tones located the farthest (x axis M = 1.19, SD = 10.19, y axis M = − 2.42, SD = 10.5, radial distance = 2.69). These results thus invalidate the centrality hypothesis, since the most stable tones were not located closest to the axes’ origin.

Finally, we examined how tonal stability associates with vertical and horizontal orientations, separately. Participants’ responses were analyzed with two mixed linear models, one for the localizations on the x axis and one for localizations on the y axis. The mixed linear models’ variables of Experiments 1-3 are presented in Table 1. In contrast to Hypothesis #2, according to which stable scale degrees should be located lower in space, participants chose significantly higher spatial positions for more stable scale degrees (stable diatonic vs. unstable: t = − 3, *p* = 0.0034, stable diatonic vs. chromatic: t = − 3.41, *p* = 0.0011). Participants also localized more stable scale degrees in more left-hand positions (stable diatonic vs. unstable: t = 2.35, *p* = 0.02, stable diatonic vs. chromatic: t = 3.19, *p* = 0.002). Finally, participants’ responses for both axes were not affected by musical experience (all interactions’ *p*s > 0.05).

**Table 1:** Fixed effects results in Experiments 1, 2 and 3. Bold values represent significant fixed effects or interactions.

Experiment	Dependent variable	Fixed effect	Estimate	Std. error	Df	t/z value	*p* value
1	Y	(Intercept)	52.60	2.06	47.41	25.51	< 0.001
		Category: unstable diatonic	− 4.56	1.52	110.24	− 3.00	**0.003**
		Category: chromatic	− 5.61	1.65	72.38	− 3.41	**0.001**
		Mode: minor	0.07	1.44	357.76	0.05	0.96
		Group: non-musicians	− 1.41	2.85	47.85	− 0.50	0.62
		Category: unstable diatonic X	0.61	1.78	7752.65	0.34	0.73
		Mode: minor					
		Category: chromatic X	2.44	1.78	7752.65	1.37	0.17
		Mode: minor					
		Category: unstable diatonic X	2.59	2.12	114.57	1.22	0.22
		Group: non-musicians					
		Category: chromatic X	0.70	2.29	74.29	0.31	0.76
		Group: non-musicians					
		Mode: minor X	− 1.40	2.01	380.31	− 0.70	0.48
		Group: non-musicians					
		Category: unstable diatonic X	0.54	2.49	7752.65	0.22	0.83
		Mode: minor X					
		Group: non-musicians					
		Category: chromatic X	0.68	2.49	7752.65	0.27	0.78
		Mode: minor X					
		Group: non-musicians					
	X	(Intercept)	48.06	1.45	62.23	33.03	< 0.001
		Category: unstable diatonic	3.22	1.37	162.65	2.35	**0.020**
		Category: chromatic	5.67	1.78	63.45	3.19	**0.002**
		Mode: minor	0.97	1.31	7792.12	0.73	0.46
		Group: non-musicians	− 0.36	2.02	63.39	− 0.18	0.86
		Category: unstable diatonic X	− 0.88	1.70	7792.12	− 0.52	0.61
		Mode: minor					
		Category: chromatic X	− 2.74	1.70	7792.12	− 1.61	0.11
		Mode: minor					
		Category: unstable diatonic X	− 1.66	1.91	169.90	− 0.87	0.39
		Group: non-musicians					
		Category: chromatic X	− 3.99	2.46	64.67	− 1.62	0.11
		Group: non-musicians					
		Mode: minor X	0.14	1.84	7792.12	0.08	0.94
		Group: non-musicians					
		Category: unstable diatonicX	0.47	2.38	7792.12	0.20	0.84
		Mode: minor X					
		Group: non-musicians					
		Category: chromatic X	1.99	2.38	7792.12	0.84	0.40
		Mode: minor X					
		Group: non-musicians					
2	RTs	(Intercept)	642.13	16.76	38.66	38.31	< 0.001
		Congruence: incongruent	0.78	8.13	54.85	0.10	0.92
		Group: non-musicians	59.28	23.74	38.91	2.50	**0.017**
		Modality: visual	− 139.37	22.79	39.53	− 6.12	** < 0.001**
		Congruence: incongruent X	12.83	11.67	58.31	1.10	0.28
		Group: non-musicians					
		Congruence: incongruent X	9.60	7.02	20,540.88	1.37	0.17
		Modality: visual					
		Group: non-musicians X	36.98	32.27	39.73	1.15	0.26
		Modality: visual					
		Congruence: incongruent X	5.00	10.24	20,573.48	0.49	0.63
		Group: non-musicians X					
		Modality: visual					
	Accuracy	(Intercept)	3.37	0.29		11.79	< 0.001
		Congruence: incongruent	− 0.30	0.13		− 2.23	**0.026**
		Modality: visual	− 0.37	0.28		− 1.33	0.185
		Group: non-musicians	− 1.14	0.39		− 2.90	**0.004**
		Congruence: incongruent X	0.01	0.16		0.05	0.96
		Modality: visual					
		Congruence: incongruent X	0.10	0.16		0.58	0.56
		Group: non-musicians					
		Modality: visual X	0.79	0.39		2.05	**0.041**
		Group: non-musicians					
		Congruence: incongruent X	− 0.04	0.20		− 0.22	0.83
		Modality: visual X					
		Group: non-musicians					
3	RTs	(Intercept)	631.67	26.95	26.19	23.44	< 0.001
		Congruence: congruent	24.78	7.25	42.03	3.42	**0.001**
		Group: non-musicians	64.01	38.13	26.23	1.68	0.11
		Modality: visual	− 209.27	25.77	26.84	− 8.12	** < 0.001**
		Congruence: congruent X	− 8.12	10.38	43.89	− 0.78	0.49
		Group: non-musicians					
		Congruence: congruent X	− 21.65	6.83	14,561.35	− 3.17	**0.002**
		Modality: visual					
		Group: non-musicians X	− 58.81	36.46	26.87	− 1.61	0.12
		Modality: visual					
		Congruence: congruent X	7.54	9.79	14,571.60	0.77	0.44
		Group: non-musicians X					
		Modality: visual					
	Accuracy	(Intercept)	3.18	0.34		9.32	< 0.001
		Congruence: congruent	− 0.22	0.18		− 1.21	0.23
		Group: non-musicians	− 0.79	0.47		− 1.66	0.096
		Modality: visual	0.87	0.18		4.90	** < 0.001**
		Congruence: congruent X	0.01	0.23		0.06	0.95
		Group: non-musicians					
		Congruence: congruent X	0.26	0.26		1.01	0.31
		Modality: visual					
		Group: non-musicians X	1.48	0.26		5.60	** < 0.001**
		Modality: visual					
		Congruence: congruent X	− 0.44	0.37		− 1.18	0.24
		Group: non-musicians X					
		Modality: visual					

#### Experiments 2 and 3

**Figure 5:**
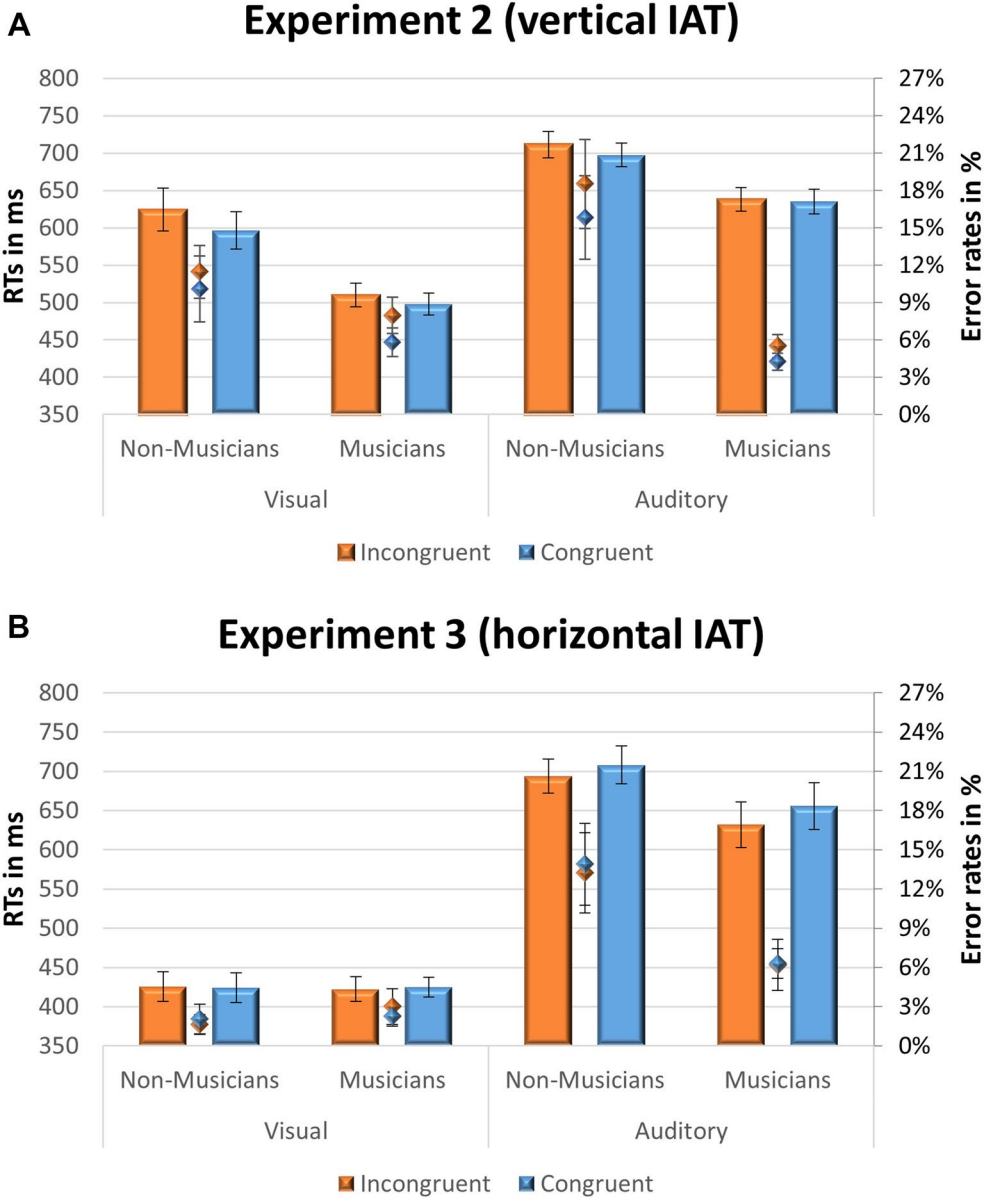
(**A**) Mean reaction times (bars) and error rates (diamonds) in Experiment 2 (IAT probing the vertical axis). (**B**) Mean reaction times (bars) and error rates (diamonds) in Experiment 3 (IAT probing the horizontal axis), for congruent (blue) and incongruent (red) blocks of trials, as a function of modality (visual, auditory), and musical experience (musicians, non-musicians). Congruency was determined according to Experiment 1 results (e.g., tonally stable degrees were paired with upper and left-hand positions and tonally unstable degrees were paired with lower and right-hand positions). Line bars represent the standard errors.

The participants who took part in Experiment 1 also participated in the two cross-modal versions of the Implicit Association Test (IAT)^35^. These two IAT experiments were designed to measure implicit associations between tonal stability (stable/unstable auditory stimuli) and vertical height (high/low circle location, 40 participants, Exp. 2), or laterality (right-hand/left-hand positions, 28 participants, Exp. 3). The congruency conditions in these IATs were specified following the results obtained in Experiment 1. Accordingly, in Experiment 2, blocks of trials in which response keys to stable auditory stimuli were paired with the higher-location circle, and response keys to unstable stimuli were paired with the lower-location circle were labelled “congruent”. Likewise, in Experiment 3, blocks of trials in which response keys to stable auditory stimuli were paired with the left-hand position, and response keys to unstable stimuli were paired with right-hand position were labelled “congruent”. Mean reaction times and error rates for the congruent and incongruent conditions of Experiments 2 and 3 are presented in Fig. 5. For each IAT experiment, reaction times were analyzed with mixed linear models and accuracy rates with generalized mixed linear models (see Table 1).

On the implicit test probing the vertical axis (Exp. 2), participants were more accurate (z = − 2.226, *p* = 0.026) but not faster (t = 0.096, *p* = 0.924) in congruent blocks than incongruent blocks. However, further Bayesian analysis revealed moderate evidence for H1 in RTs (BF_10_ **=** 2.94, error% < 0.001, see supplementary materials B for further details). The congruency effect on accuracy was similar for musicians and non-musicians (*p* = 0.56) and for responses to visual and auditory stimuli (*p* = 0.96).

The results for the implicit test probing the horizontal axis (Exp. 3) did not show a consistent pattern. Participants were faster on *incongruent* blocks than on congruent blocks (t = 3.42, *p* = 0.0014), but this effect was significant only in responses to auditory stimuli (pairwise t = − 3.0, *p* = 0.005), and not in responses to visual stimuli (pairwise t = − 0.51, *p* = 0.61), resulting in a significant interaction with modality, t = − 3.17, *p* = 0.0015. In addition, the congruency effect was not significant on accuracy (Z = − 121, *p* = 0.23). Further Bayesian analysis revealed moderate evidence for H0: BF_01_ = 4.91, error% = 0.005 (see supplementary materials B for further details).

### Physical size

#### Experiments 4 and 4A

Using a variant of the probe-tone method^34^, we had 40 participants (20 musicians) who did not participate in Experiments 1–3, match each of the chromatic scale degree to a circle’s size. Each participant was presented with a row of seven circles ranging from smallest to largest, either from right to left or from left to right (between participants).

All main effects and interactions involving tonal stability, group and circles’ orientation were significant (see Table 2 for descriptive statistics, and Table 3 for mixed-linear models results). Further analyses aimed at clarifying these interactions revealed that only musicians who were presented with the large circle on the left showed a consistent pattern: they matched larger circles to more stable the tones, (pairwise t = 5.94, *p* < 0.001, and pairwise t = 6.82, p < 0.001, for stable diatonic vs. unstable diatonic, and unstable diatonic vs. chromatic, respectively), thus partially validating Hypothesis #4. The remaining participants (i.e., both groups of non-musicians as well as musicians who were presented with the large circle on the right) showed no consistent association between size and tonal stability. Before attempting to explain these unexpected results, we sought to replicate them.

**Table 2:** Mean (in bold) ± SD of size matchings in Experiments 4 and 4A as a function of musical experience group (musicians/non-musicians) and circles’ direction (large circle to small circle from right to left, or from left to right).

Experiment	Group	Circles direction	Stable diatonic	Unstable diatonic	Chromatic
4	Musicians	Left–right	**5.43** ± 1.74	**3.71** ± 1.76	**3.46** ± 1.73
		Right–left	**4.46** ± 1.75	**4.27** ± 1.7	**4.27** ± 1.95
	Non-musicians	Left–right	**3.96** ± 1.7	**3.91** ± 1.75	**3.9** ± 1.68
		Right–left	**4.07** ± 1.73	**4.02** ± 1.77	**3.96** ± 1.77
4A	Musicians	Left–right	**4.58** ± 1.6	**3.98** ± 1.72	**4.03** ± 1.67
		Right–left	**4.44** ± 1.51	**4.33** ± 1.67	**4.22** ± 1.63

**Table 3:** Fixed effects results in Experiments 4, 4A and 5. Bold values represent significant fixed effects or interactions.Bold values represent significant fixed effects or interactions.

Experiment	Dependent variable	Fixed effect	Estimate	Std. error	Df	t/z value	*p* value
4	Size	(Intercept)	5.50	0.25	49.85	21.92	< 0.001
		Category: unstable diatonic	− 1.60	0.31	50.90	− 5.17	** < 0.001**
		Category: chromatic	− 2.20	0.37	46.38	− 5.94	** < 0.001**
		Group: non-musician	− 1.48	0.35	49.85	− 4.16	** < 0.001**
		Large circle position: right	− 0.90	0.35	49.85	− 2.55	**0.014**
		Mode: minor	− 0.14	0.16	7560.01	− 0.91	0.36
		Category: unstable diatonic X	1.55	0.44	50.90	3.55	**0.001**
		Group: non-musician					
		Category: chromatic X	2.10	0.52	46.38	4.03	** < 0.001**
		Group: non-musician					
		Category: unstable diatonic X	1.28	0.44	50.90	2.93	**0.005**
		Large circle: right					
		Category: chromatic X	1.89	0.52	46.38	3.63	**0.001**
		Large circle: right					
		Group: non-musician X	0.91	0.50	49.85	1.82	0.075
		Large circle: right					
		Category: unstable diatonic X	− 0.21	0.20	7560.01	− 1.06	0.29
		Mode: minor					
		Category: chromatic X	0.48	0.20	7560.01	2.43	**0.015**
		Mode: minor					
		Group: non-musician X	0.03	0.22	7560.01	0.12	0.90
		Mode: minor					
		Large circle: right X	− 0.11	0.22	7560.01	− 0.51	0.61
		Mode: minor					
		Category: unstable diatonic X	− 1.26	0.62	50.90	− 2.04	**0.046**
		Group:non-musician X					
		Large circle: right					
		Category: chromatic X	− 1.91	0.74	46.38	− 2.59	**0.013**
		Group:non-musician X					
		Large circle: right					
		Category: unstable diatonic X	0.20	0.28	7560.01	0.73	0.47
		Group: non-musician X					
		Mode: minor					
		Category: chromatic X	− 0.44	0.28	7560.01	− 1.57	0.12
		Group: non-musician X					
		Mode: minor					
		Category: unstable diatonic X	0.45	0.28	7560.01	1.60	0.11
		Large circle: right X					
		Mode: minor					
		Category: chromatic X	− 0.27	0.28	7560.01	− 0.98	0.33
		Large circle: right X					
		Mode: minor					
		Group: non-musician X	0.29	0.31	7560.01	0.95	0.34
		Large circle: right X					
		Mode: minor					
		Category: unstable diatonic X	− 0.48	0.40	7560.01	− 1.20	0.23
		Group: non-musician X					
		Large circle: right X					
		Mode: minor					
		Category: chromatic X	0.25	0.40	7560.01	0.64	0.52
		Group: non-musician X					
		Large circle: right X					
		Mode: minor					
4a	size	(Intercept)	4.54	0.18	17.92	25.13	< 0.001
		Category: unstable diatonic	− 0.54	0.17	35.06	− 3.16	**0.003**
		Category: chromatic	− 0.58	0.19	24.36	− 3.11	**0.005**
		Large circle position: right	− 0.13	0.16	4204.00	− 0.80	0.42
		Mode: minor	0.09	0.15	4204.00	0.63	0.53
		Category: unstable diatonic X	0.53	0.20	4142.00	2.65	**0.008**
		Large circle: right					
		Category: chromatic X	0.39	0.20	4197.00	1.97	**0.049**
		Large circle: right					
		Category: unstable diatonic X	− 0.21	0.19	4204.00	− 1.14	0.26
		Mode: minor					
		Category: chromatic X	0.10	0.19	4204.00	0.52	0.60
		Mode: minor					
		Large circle: right X	− 0.03	0.22	4204.00	− 0.16	0.87
		Mode: minor					
		Category: unstable diatonic X	0.01	0.28	4204.00	0.03	0.98
		Large circle: right X					
		Mode: minor					
		Category: chromatic X	− 0.16	0.28	4204.00	− 0.57	0.57
		Large circle: right X					
		Mode: minor					
5	RTs	(Intercept)	536.45	20.07	15.15	26.73	< 0.001
		Congruence: congruent	10.72	9.63	19.62	1.11	0.28
		Group: non-musicians	− 29.14	12.52	17.17	− 2.33	**0.032**
		Modality: visual	7.22	27.61	15.20	0.26	0.80
		Congruence: congruent X	7.42	6.76	9898.58	1.10	0.27
		Group: non-musicians					
		Congruence: congruent X	− 17.81	13.32	20.07	− 1.34	0.20
		Modality: visual					
		Group: non-musicians X	− 21.49	17.23	17.27	− 1.25	0.23
		Modality: visual					
		Congruence: congruent X	− 4.96	9.35	9900.43	− 0.53	0.60
		Group: non-musicians X					
		Modality: visual					
	Accuracy	(Intercept)	2.47	0.37	6.72		< 0.001
		Congruence: congruent	− 0.24	0.18	− 1.36		0.17
		Group: non-musicians	− 0.48	0.50	− 0.96		0.34
		Modality: visual	− 0.16	0.23	− 0.70		0.49
		Congruence: congruent X	0.16	0.24	0.66		0.51
		Group: non-musicians					
		Congruence: congruent X	− 0.04	0.18	− 0.22		0.83
		Modality: visual					
		Group: non-musicians X	0.97	0.32	3.05		**0.002**
		Modality: visual					
		Congruence: congruent X	− 0.26	0.25	− 1.07		0.29
		Group: non-musicians X					
		Modality: visual					

Since the critical effect was found only in musicians (who possess conceptual musical knowledge), we conducted a follow-up experiment (Exp. 4A) with 10 additional musicians as participants. Unlike in the main experiment, the direction of the circles’ presentation was manipulated within participant. Each musician underwent two experimental sessions, one with larger circles presented to the left of smaller circles, and the other with larger circles presented to the right of smaller circles (which order came first was counterbalanced across participants). We replicated the finding that musicians associate larger circles to more stable tones only when larger circles are presented on the left, pairwise t = 3.21 *p* = 0.042, and pairwise t = 2.885, *p* = 0.045, for stable diatonic vs. unstable diatonic, and unstable diatonic vs. chromatic, respectively), but not when they are presented on the right (both *p*s > 0.05).

#### Experiment 5

Seventeen participants (8 musicians) underwent an IAT experiment associating circle size and tonal stability (Exp. 5). Participants were equally fast and accurate, irrespective of whether the response to the stable auditory stimuli was paired with the larger or the smaller circle. Further Bayesian analysis revealed moderate evidence for H0 on RTs (BF_01_ = 3.45, error% = 0.005), and no clear evidence for either H0 or H1 on accuracy (BF_01_ = 1.03, error% = 0.006). Notice, however, that the sample size selection analysis found that 12 participants are the minimum required sample size to reach congruency effect power of 0.80 for accuracy rates.

## Discussion

Two main findings stand out from this study. First, participants—musicians as well as non-musicians—systematically associated aspects of physical space with tonal stability: scale degrees’ stability ratings strongly correlated with their perceived distance from the tonic, and tonally stable scale degrees were localized higher and to the left of unstable degrees. Second, associations between tonal stability and spatial dimensions substantially differed from those suggested by the metaphors of centricity and gravity prevalent in musical discourse. Stable scale degrees were explicitly and implicitly associated with higher, rather than with lower spatial positions (in contrast to Hypothesis #2), and with left-hand, rather than with central positions (in contrast to Hypothesis #3). Notably, musicians (who possess explicit conceptual knowledge of tonality) showed spatial mappings that contrasted strikingly with those implied by the metaphors they habitually use, suggesting that they do not rely on their conceptual knowledge of tonality in associating tonality with spatial dimensions. In fact, musicians mapped tonal relationships onto space similarly to non-musicians, who possess no conceptual knowledge of tonality.

### Vertical position

The finding that stable scale degrees were localized higher than unstable ones was particularly robust. It occurred in musicians and non-musicians alike, in both explicit (Exp.1) and implicit (Exp. 2) tasks, thus indicating that the association is not mediated by reflective conceptual processes. However, this finding sharply contrasts with our hypothesis (#2): clearly, participants systematically intuited a relationship between tonal stability and vertical position; yet, just as clearly, that intuition had little to do with established spatial metaphors associated with tonality (gravity, cadences “falling”, closural melodic lines descending towards the tonic). What, then, might be its source?

We speculate that this mapping can be explained, at least in part, via a conceptual metaphor of a considerably wider scope than the domain-specific metaphors for tonality: the “good is up” metaphor^4–8,10,11^. In line with this conjecture, previous research has linked tonal stability to valence^17–19^, and in particular, we^39^ recently reported that more stable scale degrees are associated with happier facial expressions both explicitly and implicitly, using the same paradigms as in the present study. Thus, the association between tonal stability and vertical position found here may be mediated by emotional valence: tonally stable is good (i.e., emotionally positive); ergo, tonally stable is up. This interpretation is further supported by an increasing number of empirical studies suggesting that cross-modal correspondences may be mediated by emotion in musical contexts, such that a musical feature and a non-auditory feature (e.g., a specific color or smell) are related via shared association with the same emotion (^40–43^; see also^33^, for a comparable interpretation of the association of tonal stability with visual brightness).

### Lateral position

The second consistent correspondence emerging from this study involves lateral position: stable scale degrees were localized to the left of unstable degrees, for both musicians and non-musicians. However, the correspondence was observed only in the explicit task (Exp. 1), with the implicit task (Exp. 3) showing a weak trend in the opposite direction (i.e., participants responded faster when stable auditory stimuli were paired with righthand responses).

Unlike the vertical location association, the lateral association may be explained by music-specific connotations. Left-hand localization of tonal stability may be mediated by pitch height. First, tones lower in pitch are associated with left-side spatial positions—though mainly by musicians, since the association probably stems from the structure of musical keyboards (see^32^ for research review). Second, in tonal music, the bass voice, lowest in pitch, is in several respects the most stable: it tends to be metrically stable, longer in duration, and less ornamented than the upper voice^44,45^. Thus, the laterality association of tonally stable notes may be mediated by pitch height: stability is associated with low pitch, itself associated left-hand position.

This interpretation nicely dovetails the results of Experiments 4 and 4A, which examined the correspondence between tonal stability and size. For musicians, stabler scale degrees were associated with larger objects—but only when larger objects were situated to the left. Notably, larger size has been repeatedly associated with lower auditory pitch in cross-modal research (see^31,32^ for empirical reviews). Our results thus show that tonally stabler notes are larger, but only when associated with “lower” (“larger”) pitch via its left-hand lateral position on musical keyboards.

Taken together, our findings indicate that the association of tonal stability and left-hand location is primarily explicit and that it may be derived from musical practice and its related conceptual knowledge. By contrast, the association between tonal stability and vertical location, which is shared by musicians and non-musicians both explicitly and implicitly, is independent of music-specific knowledge or practice and is likely to be mediated by implicit associations between emotional valence and spatial height.

## Conclusion

Beyond specific correspondences, the most noteworthy finding of this study is its broadest: listeners map tonal hierarchy onto perceived physical space. These mappings, which differ from those established by musical discourse, are mostly shared by musicians and non-musicians, suggesting that they are independent of musical expertise and conceptual music-theoretical knowledge. Strikingly, participants with no conceptual musical knowledge, asked to map tonal stimuli spatially, intuitively localized these stimuli such that their spatial distances closely correlated with their tonal stability differences. Furthermore, the most robust correspondence found here, between tonal stability and vertical position, emerged via an implicit paradigm (IAT) as well, possibly reflecting an automatic, involuntary association between the two dimensions.

The significance of these findings is threefold. For music, they suggest one path through which musical structure—often considered to be abstract and non-referential—may refer to perceived physical domains. For cross-modal research they present a novel type of correspondence (see also^33^), between abstract syntactical features and concrete perceptual dimensions, across modalities. These findings thus demonstrate that syntactical structures may possess a connotative, semantic function—at least, but perhaps not solely, in the musical domain.

Finally, the present study provides a novel perspective on the notion that spatial mappings underlie the cognition of abstract domains—and on the relationship between such mappings and conceptual metaphors. Here, spatial mappings were similar for participants with or without conceptual knowledge of the target domain (tonality); furthermore, the former group (musicians) mapped tonality onto space in ways that are incongruent with the verbal metaphors they use to describe it. Thus, non-verbal spatial mappings of abstract domains may be independent of the spatial metaphors describing that domain.

## Materials and methods

Ethical clearance was obtained for experimental data collection by Tel-Aviv University Institutional Review Board. Methods were carried out in accordance with the ethical guidelines and informed consent was obtained from all the participants.

### Sample size selections

For Experiments 1 and 4, we calculated the sample size required to observe a significant effect of tonal hierarchy, based on the study by Maimon et al. (ref.^33^; Exp. 1, brightness session). Since the present study adopted the same procedure, we simulated the different sample sizes with “simr”—a power analysis package for r, designed to interoperate with the lme4 package for LMMs^46^. We found that the minimum required sample size to reach the power 0.80 were 15 participants (see supplementary materials B for simulation output graphs). For Experiments 2, 3 nd 5 we calculated sample size based on Maimon et al. (ref.^33^; Exp. 2, IAT of tonal stability and visual brightness). We found that for reaction times that the minimum required sample size to reach the power 0.90 were 30 participants, and for accuracy rates 12 participants (simulating generalized mixed linear model).

### Apparatus

The stimuli were presented on a computer with an Intel Core i7 CPU 920 processor, 2.67 GHz speed, and 2.98 GB RAM memory. Auditory stimuli were delivered through Sennheiser 210HD precision headphones. These were connected to the computer by a Terratec Producer, Phase 24 sound card. Stimulus loudness was measured through Brüel and Kjær type 2232 noise meter, measuring A-weighted dB. The computer screen was a 17-inch Lenovo LCD, with a screen refresh frequency of 85 Hz and 1024 × 768-pixel resolution. The experiments were programmed and run using Matlab R2014b with psychtoolbox^47^.

### Experiment 1

#### Participants

Forty undergraduate and graduate students from Tel-Aviv University, 17 musicians (9 female, mean age = 20.93, SD = 2.74) and 20 non-musicians (12 female, mean age = 23.59, SD = 2.13) served as participants. The musicians had an average of 11.25 (SD = 3.4) years of musical experience (with a minimum of 7 years) and an average of 7.18 (SD = 2.09) years of music theory studies (with a minimum of 5 years). They all currently played and performed music. The non-musicians had an average of 2 (SD = 1.41) years of musical experience (with a maximum of 3 years in childhood), no music theory education, and none of them currently played music. All participants participated in the experiment for monetary credit (25$ for two sessions of 1 h, reported as Experiments 1 and 2).

#### Auditory stimuli

Pitch height is known to be strongly associated with visual space^48^. In order to minimize pitch-height effects, all auditory stimuli (both contexts and probes) were created using Shepard tones^49^, Shepard tones are tones with a specific pitch chroma (pitch-class), sounded in 5 octaves simultaneously. Following the standard protocol presented in^49^, we created the tones using a loudness envelope across the frequency range of 77.8–2349 Hz, with partials uniformly decreasing in intensity toward low and high frequencies. As a result, Shepard tones create a clear perception of pitch chroma (pitch class—All pitches an octave (or its multiples) from each other belong to the same pitch-class (or share the same pitch chroma quality). For instance, the pitches A1 (F0 = 55 Hz), A2 (110 Hz), A3 (220 Hz), A4 (440 Hz), etc., all belong to the pitch class denoted by the note-name "A".)but an ambiguous pitch height: the specific octave in which a pitch is perceived—which partial in a given tone represents its pitch height for a listener—is ambiguous and determined contextually. All stimuli were sounded in 71 dB.

Each trial consisted of a chord or a chord sequence which established a sense of tonal key—the “context element”—followed by a single tone out of the 12 chromatic tones in western tonality—the “probe”. There were eight possible contexts (see audio examples in supplementary materials A): two context types (a triad, the first, third and fifth degrees played simultaneously, presumably perceived as a tonic chord, and a IV-V-I cadence, a sequence of three chords which imply a strong notion of closure in the tonal system). Each of these two types was played in four keys (G major, G minor, D-Flat major and D-Flat minor). There were 12 possible probes: the 12 tones of the chromatic scale (12 notes from C to B). On each trial, a context was played for 0.5 s when it was a tonic chord, and for 0.5 s for each chord followed by a silence of 0.25 s when it was a cadence. Then, following a silence of 1 s, a probe tone was played for 0.5 s. Sound examples S1–S4 in supplementary materials A present four examples of a context followed by a probe. In Sound example S1, a context (a cadence) in D-flat major is followed by a stable probe (the tonic note). In Sound Example S2 the same context is followed by an unstable probe. In Sound Examples S3 and S4 contexts in G major are followed by stable and unstable probes, respectively.

The experiment consisted of 18 blocks. The first two blocks served as practice. The contexts for these blocks were quasi-randomly drawn from the eight possible contexts and consisted of either a cadence or a triad, in either G or D Flat (counterbalanced across subjects). They were followed by 16 experimental blocks, two for each of the eight possible contexts, randomly mixed. The same context was used throughout any given block of trials. Each block consisted of 14 consecutive trials. The first two trials served as practice, using two randomly selected probes. In the following 12 experimental trials, the 12 chromatic tones, randomly mixed, were used as probe tones.

#### Procedure

The experiment was conducted in a dark sound-attenuating room. Participants were seated 50 cm from the computer screen. Through the whole block, two dashed-line axes (12 cm each), were presented at the middle of the screen. On each trial, participants were instructed to listen to the context followed by the probe. Simultaneously with the auditory probe, a circle (1 cm diameter) appeared at the intersection of the 2 axes. Participants were asked to move the circle to the location that best matched the subjective judgment or “feeling,” evoked by the probe tone (the circle was the cursor and participants moved it and clicked at the selected location).

The experimenter underscored the subjective and intuitive character of the task. She instructed participants to choose whichever strategy they felt was the most suitable to perform the tasks, yet to be consistent and use the same strategy throughout the experiment. Additionally, participants were instructed to use the whole 2-dimensional space and to avoid placing the circle in the same quadrant in all trials.

After each block, participants were asked about their confidence in the judgments they had provided. Our objective was to examine whether mode and musical expertise (whether participants had professional musical training) affected how confident participants felt about their judgments. The text "How confident were you with the ratings of the last block?" appeared on the screen and participants provided a 1–7 rating by pressing the appropriate numeral on the upper row of the keyboard. Then, a 10-s. white noise (71 db) was heard, followed by a count down from 5 to 1. Participants then proceeded to the next block by pressing any key, after a self-pace break.

All written and oral instructions were presented in Hebrew.

### Experiment 2

#### Participants

Forty undergraduate and graduate students from Tel-Aviv University (20 musicians) who participated in Experiment 1. The order of the sessions was counterbalanced with a mean of 9.72 (SD = 5.18) days between sessions (and a minimum of 1 week).

#### Auditory stimuli

In this experiment, four short auditory stimuli (two stable and two unstable) were designed to be comparable to the auditory stimuli in Experiment 1 (i.e., context and probe, for auditory examples and graphical representations, see supplementary materials A). Since responses in this experiment were speeded, we created a shorter and faster context consisting of a half-cadence (IV-V46-35)—a chord sequence suggesting a subsequent resolution by a stable chord or tone. Each chord of the context sequence lasted for 250 ms, with a 250 ms silence apart. Since the IAT paradigm requires a speeded classification between two levels, we used the two probes that received the highest and lowest space ratings in Maimon et al.^33^ (Exp. 1). Therefore, this context was followed by a 250 ms tone, either the tonic note (scale-degree 1, a tonally stable stimulus) or the raised subdominant (4#, a tonally unstable chromatic note). To minimize possible short-term memory effects, the cadence (played in a three-voice texture) was designed such that neither of the final probe tones (tonic, raised 4th) was included in the chords preceding it. The final tone was octave-doubled, and the pitch direction (up/down) between the last chord in the cadence and the following tone was controlled. Additionally, each stimulus type (stable/unstable) was presented in two keys (C major and D-flat major), one stimulus ended with rising pitch height and one with a falling pitch height. Stimuli were sounded in a piano timbre generated by Notion 6 music notation software. Sound Examples 2a and 2b in supplementary materials A demonstrate stable and unstable auditory stimuli used in this experiment.

#### Visual stimuli

The visual stimuli consisted of two grey circles, one with a presented 30° higher in the visual field and one 30° lower in the visual field.

#### Procedure

Congruent and incongruent trial sequences examples are presented in Fig. 1C. On each trial, participants were presented with a unimodal stimulus (either auditory or visual). They were asked to classify it using one of two keys (i.e., K or D). For the visual stimuli, one key was assigned to the circle presented spatially high, and another to the spatially low circle. For the auditory stimuli, one key was assigned to a stable progression, and the other to an unstable progression. Note that the experimenter did not mention any of these adjectives (up, down, high, low, stable or unstable etc.) but instead referred to the visual and auditory stimuli as type K and type D according to the participant’s key assignment in the practice blocks. The experiment consisted of 24 blocks. All stimuli were presented in all blocks. Each block consisted of 28 consecutive trials. The first four trials served as practice trials and included one stimulus of each type (auditory stable, auditory unstable, spatially high and spatially low). In the following 24 experimental trials, the two visual stimuli were presented 6 times each, and the four auditory stimuli (stable and unstable in C and C# keys) were presented 3 times each, all randomly mixed. Each of the four possible stimulus-key pairings (2 congruent and 2 incongruent pairings) were presented in different blocks. There were 6 blocks for each pairing, resulting in 24 experimental blocks presented in randomly mixed order. The congruency conditions in these IATs were specified following the results obtained in Experiment 1. Thus, in half of the blocks, the response pairing was congruent (i.e., the same response was associated with the stable auditory stimuli and the spatially high circle and with the unstable auditory stimuli and the spatially low circle), whereas in the remaining half of the blocks, the response pairing was incongruent (i.e., the same response was associated with the unstable auditory stimuli and the spatially high circle and with the stable auditory stimuli and the spatially low circle).

### Experiment 3

#### Participants

28 participants that underwent experiment 1 and 2 were invited for a third session approximately 4 months after the last session. 14 musicians (7 females), mean age = 22.28, SD = 3.17 and 14 non-musicians (6 females), mean age = 23.78, SD = 1.52. The musicians had an average of 12.28 (SD = 2.71) years of musical experience (with a minimum of 7 years) and an average of 6.28 (SD = 1.57) years of music theory studies (with a minimum of 5 years). All participants participated in the experiment for monetary credit (15$).

*Apparatus, stimuli, and procedure* were the same as experiment 2. Visual stimuli in this experiment included circles were presented either 30° towards the left of the screen and 30° towards the right of the screen. Importantly, to avoid Simon effect^50^, reaction keys for visual stimuli were consistent (D for the left circle, and K for the right).

### Experiment 4

#### Participants

Forty undergraduate and graduate students from Tel-Aviv University, 20 musicians (10 female) mean age = 22.82, SD = 3.21 and 20 non-musicians (15 female) mean age = 25.4, SD = 3.21 served as participants. The musicians had an average of 15.25 (SD = 3.15) years of musical experience (with a minimum of 7 years) and an average of 8.15 (SD = 3.02) years of music theory studies (with a minimum of 5 years). All participants participated in the experiment for monetary payment (15$).

*Apparatus, stimuli, and procedure* were the same as experiment 1. Visual stimuli in this experiment included 7 circles presented with varying size from small (1 cm diameter) to large (5 cm diameter). Half of the participants were presented with the small circle on the left and half with small circle on the right.

### Experiment 4A

#### Participants

10 musicians (3 females) from Tel-Aviv University who did not participate in Experiments 1–4, mean age of 24.41 SD = 4.03, years of musical experience 12 SD = 6.88, and 6.65 SD = 3.47 years of music theory studies. All participants participated in the experiment for monetary payment (25$ for the two sessions).

*Apparatus, stimuli, and procedure* were the same as experiment 4. Procedure included two experimental sessions per participant, one with the small circle presented on the right and one with the small circle presented on the left (order counter balanced).

### Experiment 5

#### Participants

17 undergraduate and graduate students from Tel-Aviv University, 8 musicians (6 females) mean age = 22.75, SD = 4.23 and 9 non-musicians (4 females) mean age = 23.88, SD = 3.92 served as participants. The musicians had an average of 11.75 (SD = 2.1) years of musical experience (with a minimum of 7 years) and an average of 5.87 (SD = 2.89) years of music theory studies (with a minimum of 4 years). All participants participated in the experiment for monetary credit (15$).

*Apparatus, stimuli, and procedure* were the same as experiment 2. Visual stimuli included a large circle (3 cm diameter) and small circle (1 cm diameter) presented at the middle of the screen.

### Statistical analyses

#### Probe-tone experiments (Experiments 1, 4 and 4A)

Independent variables included musical training (musician/non-musician) as a between-participants variable, and mode (major/minor) and tonal stability as within-participant variables. The tonal stability variable included three categories: Stable diatonic (the tonic chord members—the 1st, 3rd, and 5th degrees of the scale; that is, tones 1, 5, 8 of the 12 chromatic tones in major and 1, 4, 8 in minor), Unstable diatonic (2nd, 4th, 6th and 7th degrees of the scale: 3, 6, 10, 12 of the 12 chromatic tones in major and 3, 6, 7, 11, 12 in minor, where both options for the 7th scale-degree, raised [leading-tone] and natural, were included) and chromatic tones (non-scale degrees: 2, 4, 7, 9, 11 of the 12 tones in major and 2 ,5 ,7, 10 in minor). Dependent variables included mean position of X axis and mean positions of Y axis in Experiment 1 and size matchings in Experiments 4 and 4A.

Mixed-effect linear regression models (one for each dependent variable) included all independent variables and their interactions as fixed effects. Participants’ random slopes were fit to the data in a step-wise-step up procedure using Chi square tests (for all models’ comparisons, see model selection section in supplementary materials B). In these models, for variables with more than two levels, dummy variables are computed. Therefore, for tonal stability, which has 3 levels (stable diatonic, unstable diatonic and chromatic), we chose stable diatonic as the reference level, and two dummy variables were computed: unstable diatonic (e.g. the difference between stable diatonic and unstable diatonic), and chromatic (e.g. the difference between stable diatonic and chromatic). Accordingly, a significant effect of the unstable diatonic variable would mean that the level of the stable diatonic category was significantly different than that of unstable diatonic; similarly, a significant effect of the chromatic variable would mean that the level of the chromatic was significantly different than that of the stable diatonic. For all final mixed-linear models conducted in the present study see Table 4. Additionally, for the significant interactions, simple effects analysis was carried out on all possible comparisons between variables levels, false discovery rate (FDR) was controlled using Benjamini and Hochberg correction (BH) for multiple comparisons^51–53^ (for all comparisons conducted in the present study see supplementary materials B). LMM analyses were conducted via lme function^46^ and simple effects analysis via lsmeans function^54^, all using RStudio 1.4.1717^55^.

**Table 4:** List of the final mixed linear models in Experiments 1–5.

Experiment	Dependent variable	Final model
1	X	Y ~ tonal stability * mode * musical experience + (tonal stability + mode | participant)participant
	Y	X ~ tonal stability * mode * musical experience + (tonal stability | participant)participant
2	RTs	RT ~ congruence * musical experience * modality + (congruence + modality | participant)participant
	Accuracy	Accuracy ~ congruence * modality * musical experience + (congruence + modality | participant)participant
3	RTs	RT ~ congruence * musical experience * modality + (congruence + modality | participant)participant
	Accuracy	Accuracy ~ congruence * musical experience * modality + (congruence | participant)participant
4	Size matchings	Response ~ category * musical experience * ovaldirection * mode + (category | participant)participant
4A	Size matchings	Response ~ category * ovaldirection * mode + (category | participant)participant
5	RTs	RT ~ congruence * modality * musical experience + (congruence + modality | participant)participant
	Accuracy	Accuracy ~ congruence * musical experience * modality + (congruence + modality | participant)participant

#### IAT experiments (Experiments 2, 3 and 5)

Independent variables included musical training (musician vs. non-musician) as a between-participants variable, and congruence (congruent/incongruent) and modality (visual/auditory) as within-participant variables. Dependent variables included reaction times (correct trials only), and accuracy rates. Anticipatory responses were omitted from the analysis (i.e., RTs shorter than 150 ms.), these included 0.003% in Experiment 2, 0.005% in Experiment 3 and 0% of responses in Experiment 5. RTs mixed-effect linear regression model included all independent variables and their interactions as fixed effects. Participants’ random slopes were fit to the data in a step-wise-step up procedure using Chi square tests (see supplementary materials B section 5 for all comparisons). For accuracy rates, a mixed-effect binomial logistic regression model was fitted. For significant interactions, simple effects analysis was conducted with BH correction for multiple comparisons.

## Supplementary Information

Supplementary Information 1.

Supplementary Information 2.
