# Supplementary material for: Space oddity: musical syntax is mapped onto visual space
Source: Sci Rep. 2021 Nov 16;11:22343. doi: 10.1038/s41598-021-01393-1 (PMC8595729; doi:10.1038/s41598-021-01393-1)
Supplement: Supplementary file 1 — Supplementary Information 1. [file 41598_2021_1393_MOESM1_ESM.pdf]

Supplementary materials 1

This section is taken from Maimon et al [1]

Tonal Stability

In music theory, the term ‘tonality’ denotes a system organizing pitch relationships in a musical work, both melodically (i.e., with regard to pitch succession) and harmonically (with regard to pitch simultaneities, or ‘chords,’ and their succession), in a hierarchy of stability and closure. Importantly, for each tonal context, or musical key, pitches are organized with reference to one maximally stable pitch class, the *tonic* or *tonal center*.

What is ‘tonal stability’? Stable pitches and chords are associated with points of closure (endings of segments) in tonal music — an association underlying listeners’ perception of musical closure [2,3]. Furthermore, unstable tones tend to proceed to the nearest stable tones (but not vice versa), a distributional fact affecting listeners’ expectations [4]. Thus, unstable tones or chords tend to suggest to listeners a sense of tension and continuity, while the stable tones/chords following them suggest resolution of that tension [5]. Tonal stability also correlates with pitches’ occurrence frequency: within a tonal context, stable pitches are more frequent than instable ones. Compatibly, listeners tend to perceive stable pitches as better fitting a primed tonal context than unstable ones [6].

In Western tonal music, tonal stability is governed by musical keys. A musical key is defined by its tonic and its mode (major or minor). The tonic — which may be any of the 12 pitch classes available in Western music — is, as noted above, the maximally stable, most closural note in a given key. For each tonic, two different modes are available, major or minor, each established by a different pattern of pitch intervals between the tonic and the other constituent tones. Thus, the keys of C major and C minor share the same tonic — the pitch class C — but have different modes; that is, the relationships of some of their other constituent pitches to the tonic differs.

Each major or minor key chiefly utilizes seven out of the 12 available pitch classes, its diatonic scale degrees. Diatonic scale degrees are commonly represented as a musical scale, arranged in order of pitch height, with the tonic note presented as first (lowest) note, or scale degree 1, and the other diatonic degrees presented and numbered accordingly (2–7; see Fig. S1A, B, for scalar representations in music notation of the two keys used in our experiments — G major and D-flat major). The most stable diatonic notes are the tonic (scale degree 1) and the other constituents of the tonic triad (the chord associated with the tonic note) — scale degrees 3 and 5. Other diatonic degrees (2, 4, 6, 7) are

relatively instable, evoking tension that may be resolved when their more stable neighbors (1, 3, or 5 scale degrees) follow (5).

While simple tonal pieces (e.g., many western European folk songs) may exclusively use the seven diatonic scale degrees, more complex tonal music also applies the remaining five pitch classes (termed ‘chromatic tones’ by musicians), which are conceived (by music theorists) and perceived (by listeners; see ref 6, for review of empirical studies) as the least stable tones in a tonal context. These ‘out of key’ notes tend to evoke strong tension, resolved when their nearest diatonic (within-key) notes follow. In sum, then, musical theory, as well as music cognition research, distinguishes three main levels of tonal stability: tonal triad members (scale degrees 1, 3, 5) are the most stable; other diatonic notes (scale degrees 2, 4, 6, 7) are less stable, and tend to resolve to nearby stable scale degrees; and the remaining chromatic (out of key) notes are the least stable, strongly implying resolution by their diatonic neighbors. Figure S2 A demonstrates this hierarchy.

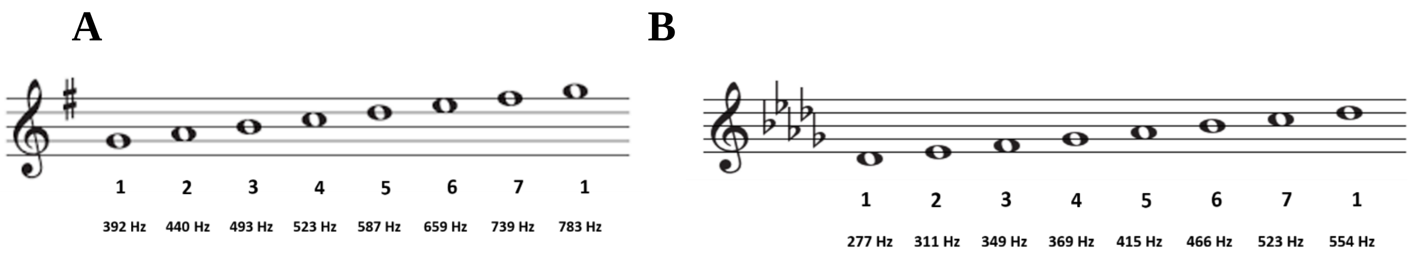

Figure S1. Musical notation of the two major scales used in Experiments 1A and 1B, G major (A), and D-flat major (B). Arabic numerals below the notes denote their scale degree identity (1–7). Fundamental frequencies (F0, in Hz) are marked below the scale degree numbers (note, however, that each scale degree can be represented by different pitch classes – notes sharing the same name, situated one or several octaves apart – e.g., A1; 55Hz; A2, 110Hz; A3, 220 Hz; A4, 440 Hz, etc).

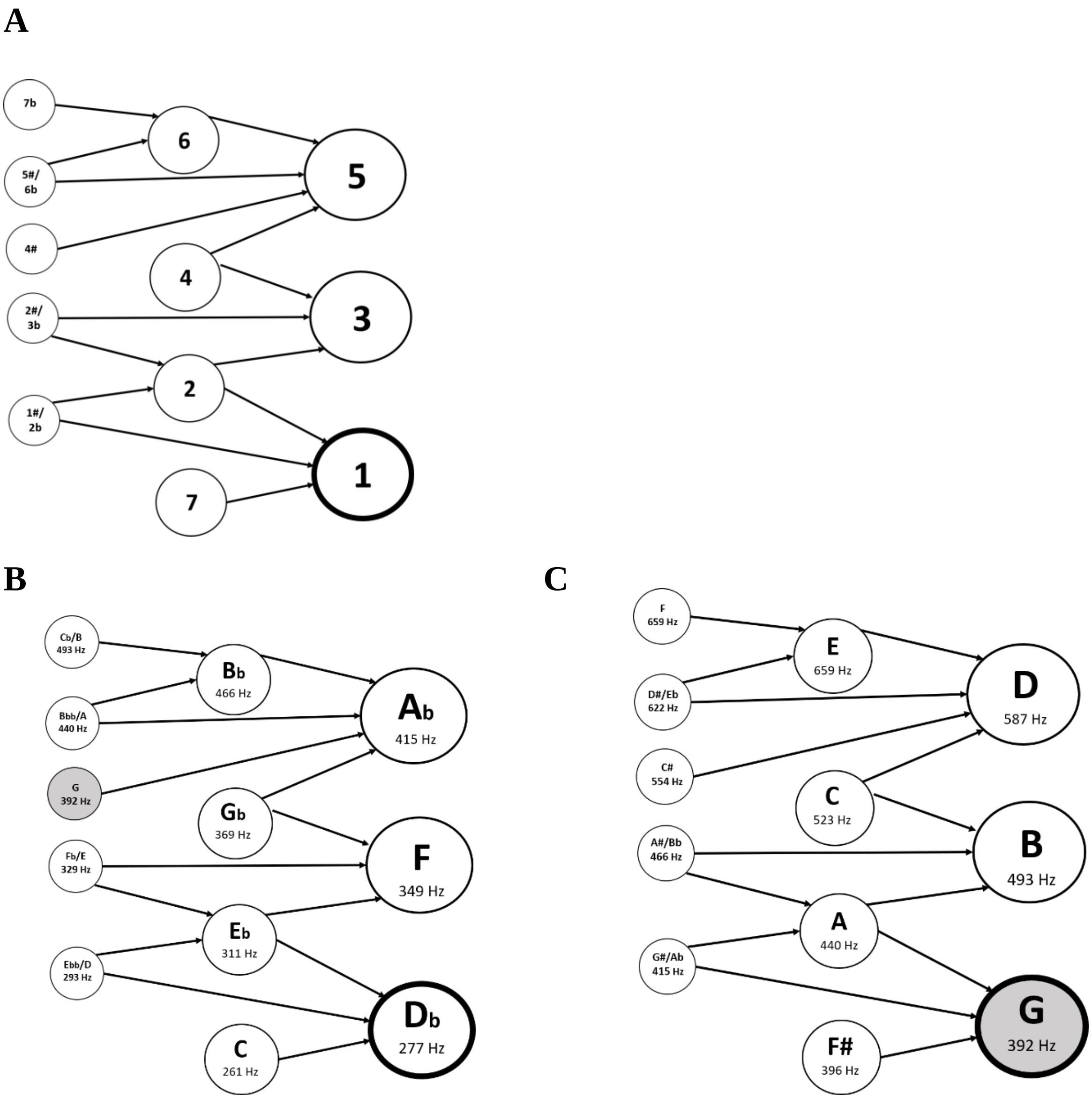

Figure S2. Visual representations of tonal hierarchy. Figure S2A presents generalized scale degree representation, while panels B and C, respectively, present these relationships in G major and D-flat major keys, replacing the scale degree numbers in A with the pitch classes representing these scale degrees in each key (fundamental frequencies are added below pitch class names, corresponding to those of pitches in Fig. S1). The larger and bolder scale degrees on the right are the most stable (stable diatonic), the middle layer presents other diatonic scale degrees — less stable yet belonging to the respective key (unstable diatonic), and the third (leftmost) layer presents the least stable tones (chromatic, 'out of key' tones). Arrows between scale degrees represent the typical motion direction between them — from an instable degree to a stable one. Note that while the tonal schema represented in 2A remains the same in both keys (2B, 2C), the representatives (i.e,. specific pitch classes) of its 'slots' (the scale degrees) radically change, with stable degrees in one key becoming instable in the other. For instance, the pitch class G (gray circles), which is the tonic note — the most stable note — in G major (2B) is an instable chromatic note in D-flat major (2C).

Importantly, the hierarchy of tonal stability is always relative to a key's tonic, such that the very same pitch may be highly stable in one key, and highly unstable in another. For instance, in the key of G major, the pitch class C-sharp/D-flat (which is a chromatic, 'out of key' note) would be extremely unstable, while G, the tonic, would be the maximally stable pitch class. In contrast, in the key of D-flat major, D-flat (the tonic note of that key) would be maximally stable, while G (here, a chromatic note) would be highly unstable. Figures 2A–C demonstrate this relativity: Fig. S2 A is a general representation of the tonal hierarchy described above. Figures S2 B and C demonstrate the same hierarchy as applied to two different keys — G major and D-flat major. Note that while the tonal schema remains the same in both keys, the representatives (i.e., specific pitch classes) of its 'slots' (the scale degrees) radically change, with stable degrees in one key becoming instable in the other.

Tonality underlies the bulk of Western music ('classical' as well as popular) since the 17th century and has been described and modeled in detail by music theorists (e.g., [7-9]). In recent decades, the psychological reality of tonality as a cognitive schema orienting the listener has been strongly established empirically (see 10-11 for research surveys). Studies applying converging experimental paradigms — explicit measurements, such as sung continuations and goodness-of-fit ratings (e.g., [12, 13, 6]), as well as implicit ones, such as musical priming (e.g., [14]) and event-related potentials (ERP; e.g., [15]) — have suggested that listeners implicitly abstract a tonal hierarchy, and the sets of melodic and harmonic expectancies it entails, closely matching those conjectured by music theorists' models.

Stimuli

Experiments 1, 4 & 4A

Audio S1. Examples of stable (A-link) and unstable (B-link) auditory stimuli used in experiments 1, 4 and 4A. Both examples consist of a cadence type context element (a sequence of three chords), followed by either a stable (A) or an unstable (B) probe.

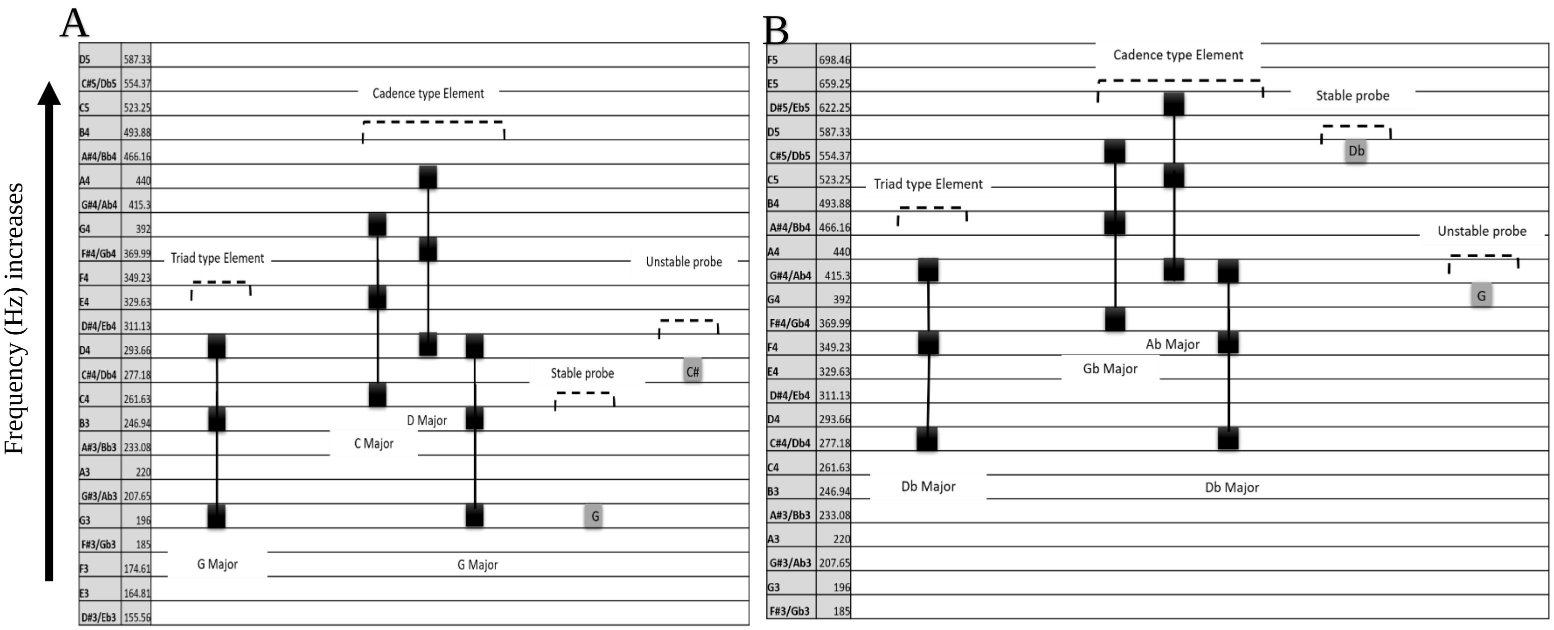

Figure S4. Schematic presentation of the auditory stimuli used in experiments 1, 4 & 4A (probe-tone method), in G Major (A) and in D flat Major (B). Each black box is a note, and the lines connecting the notes create a chord. The example shows a triad element and a cadence context element (a sequence of three chords), followed by an example of stable or unstable probe. In A, the elements are in G major key, the stable probe is the note G (the first scale degree, the most stable tone). The unstable probe is C# (the augmented forth degree, the tritone, which does not belong to the key, and is thus unstable). In B, the elements are in D flat major key, the stable probe is the note D flat (the first scale degree, the most stable tone). The unstable probe is G (the augmented forth degree, the triton, which does not belong to the scale, the least stable). Note that for the sake of clarity, each note in this schematic representation is represented by a single frequency. Stimuli used in this experiment, however, were Shepard tones [16], in which each pitch-class is created by 5 sine-tones separated by octaves.

Experiments 2, 3 and 5

A

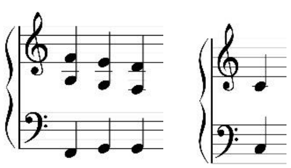

B

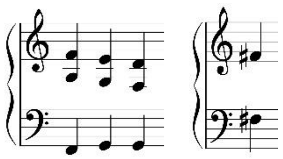

Audio S2. Examples of stable (A link) and unstable (B link) auditory stimuli used in Experiment 2, 3 & 5. Stimuli consist of a sequence of three chords followed by either a stable (A) or an unstable (B) tone.

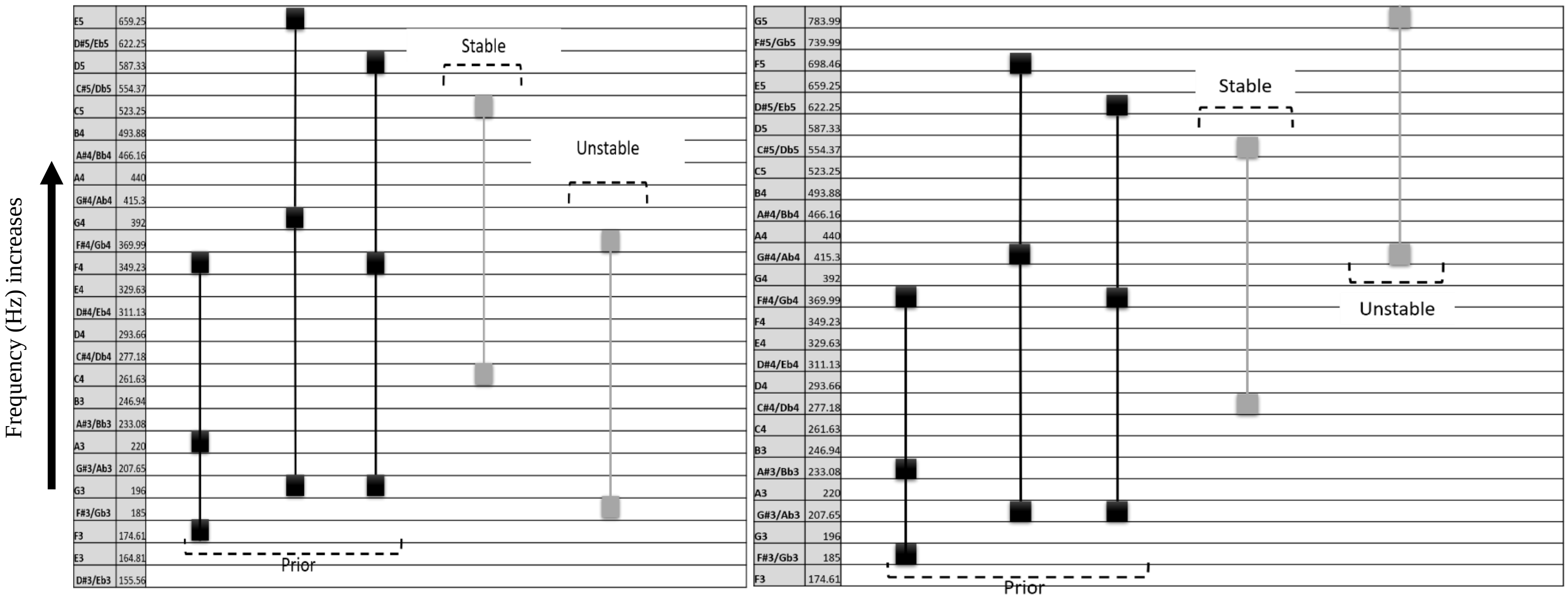

Figure S5. Schematic presentation of the auditory stimuli used in Experiment 2, 3 and 5 (IAT method), in C Major (A) and in C sharp Major (B). Each black box is a note, and the lines connecting the notes create a chord. The key in example A is C Major and consists of prior (a sequence of three chords), followed by a stable note (C, first degree of the scale, most stable) or an unstable note (F#, the raised 4th degree, least stable). The key in example B C sharp Major and consists of prior (a sequence of three chords), followed by a stable note (C sharp), first degree of the scale, most stable) and an unstable note (G, the raised 4th degree, least stable). Note that while for the sake of clarity each tone is represented here by its fundamental frequency (F0) only, stimuli consisted of harmonic tones (piano timbre).

References

1. Maimon N., Lamy D., Eitan Z., (2020) Crossmodal Correspondence Between Tonal Hierarchy and Visual Brightness: Associating Syntactic Structure and Perceptual Dimensions Across Modalities, *Multisensory Research*, ***33***(8), 805-836.
2. E. Bigand, B. Tillmann, B. Poulin-Charronnat, A module for syntactic processing in music? *Trends Cogn. Sci*. **10**, 195–196 (2006).
3. M. Boltz, Perceiving the end: Effects of tonal relationships on melodic completion. *J. Exp. Psychol. Hum. Percept. Perform.* **15**, 749–761 (1989).
4. D. Huron, *Sweet Anticipation: Music and the Psychology of Expectation*. MIT Press, Cambridge, MA, USA (2006).
5. F. Lerdahl, C. L. Krumhansl, Modeling tonal tension, *Music Percept.* **24**, 329–366 (2007).
6. C. L. Krumhansl, Tonal hierarchies and rare intervals in music cognition, *Music Percept.* **7**, 309–324 (1990).
7. F. J. Fétis, *Traité Complet de la Théorie et de la Pratique de l'Harmonie Contenant la Doctrine de la Science et de l'Art*. (Maurice Schlesinger, Paris, France, 1844/2008).
8. A. Schoenberg, Harmonielehre (Leipzig:Verlagseigentum der Universal-Edition, 1911); trans. as *Theory of Harmony*, by Roy E. Carter (Berkeley: University of California Press, 1978).
9. J.P. Rameau. Traité de l’Harmonie Reduite à ses Principes Naturels: Divisé en Quatr Livres. (Jean-Baptiste-Christophe Ballard, Paris, France, 1722).
10. C. L. Krumhansl, The cognition of tonality–as we know it today. *Journal of New Music Research*, **33**, 253-268 (2004).
11. D. Shanahan, “Musical Structure: Tonality, Melody, Harmonicity, and Counterpoint”. In *The Routledge Companion to Music Cognition*. M. Cobussen, V. Meelberg, B. Truax, Eds. (Routledge, NY, NY, USA, 2016), pp. 141-151.
12. J. C. Carlsen, Some factors which influence melodic expectancy, *Psychomusicology* **1**, 12–29 (1981).
13. L. L. Cuddy, C. A. Lunney, Expectancies generated by melodic intervals: Perceptual judgments of melodic continuity, *Atten. Percept. Psychophys*. **57**, 451–462 (1995).
14. F. Marmel, B. Tillmann, Tonal priming beyond tonics. *Music Perception: An Interdisciplinary Journal*, *26*(3), 211-221 (2009).
15. R. Granot, E. Donchin, Do re mi fa sol la ti - constraints, congruity, and musical training: an event-related brain potentials study of musical expectancies, *Music Percept*. **19**, 487–528 (2009).
16. R. N. Shepard, Circularity in judgments of relative pitch. *The Journal of the Acoustical Society of America*, **36**, 2346-2353 (1964).
