# Supplementary material for: Space oddity: musical syntax is mapped onto visual space
Source: Sci Rep. 2021 Nov 16;11:22343. doi: 10.1038/s41598-021-01393-1 (PMC8595729; doi:10.1038/s41598-021-01393-1)
Supplement: Supplementary file 2 — Supplementary Information 2. [file 41598_2021_1393_MOESM2_ESM.pdf]

1. Pairwise comparisons

1.1. Exp. 2

comparison	estimate	SE	df	t ratio	p value
musicians auditory - (non-musicians auditory)	0.11	0.03	70.7	3.6	**0.0035**
musicians auditory - musicians visual	0.02	0.03	38	0.67	0.51
musicians auditory - (non-musicians visual)	0.05	0.03	70.7	1.65	0.16
(non-musicians auditory) - musicians visual	-0.10	0.03	70.7	-3.03	**0.0103**
(non-musicians auditory) - (non-musicians visual)	-0.06	0.03	38	-2.29	0.06
musicians visual - (non-musicians visual)	0.03	0.03	70.7	1.08	0.34

Table S1: estimates, standard errors, dfs, t and p values of pairwise comparisons conducted on accuracy rates of Experiment 2 (vertical IAT) on Group X Modality interaction. P value adjustment: Benjamini Hochberg method for 6 tests

1.2. Exp. 3

comparison	estimate	SE	df	t ratio	p value
(auditory Incongruent)-(visual incongruent)	239.23	18.8	29.5	12.73	**<.0001**
(auditory Incongruent)-(auditory congruent)	-20.1	6.7	52	-3.001	**0.0049**
(auditory Incongruent)-(visual congruent)	235.79	18.8	29.5	12.55	**<.0001**
(visual incongruent)-(auditory congruent)	-259.34	18.8	29.5	-13.81	**<.0001**
(visual incongruent)-(visual congruent)	-3.44	6.7	52	-0.51	0.61
(auditory congruent)-(visual congruent)	255.9	18.8	29.5	13.62	**<.0001**

Table S2: estimates, standard errors, dfs, t and p values of pairwise comparisons conducted on reaction times of Experiment 3 (horizontal IAT) on Congruence X Modality interaction. P value adjustment: Benjamini Hochberg method for 6 tests

comparison	estimate	SE	df	t ratio	p value
(musicians auditory) – (non-musicians auditory)	0.07	0.03	42.3	2.72	**0.0189**
(musicians auditory) – (musicians visual)	-0.04	0.02	26	-1.86	0.11
(musicians auditory) – (non-musicians visual)	-0.04	0.03	42.3	-1.63	0.13
(non-musicians auditory) – (musicians visual)	-0.11	0.03	42.3	-4.06	**0.0006**
(non-musicians auditory) – (non-musicians visual)	-0.12	0.02	26	-6.03	**<.0001**
(musicians visual) – (non-musicians visual)	-0.01	0.03	42.3	-0.29	0.77

Table S3: estimates, standard errors, dfs, t and p values of pairwise comparisons conducted on accuracy rates of Experiment 3 (horizontal IAT) on Musical experience X Modality interaction. P value adjustment: Benjamini Hochberg method for 6 tests

1.3. Exps. 4 & 4a

comparison	estimate	SE	df	t ratio	p value
1stable diatonic musician left -2 unstable diatonic musician left	1.71	0.29	72	5.94	**<.0001**
1stable diatonic musician left -3 chromatic musician left	1.97	0.29	72	6.82	**<.0001**
1stable diatonic musician left -(1 stable diatonic non-musician left)	1.46	0.29	108	5.01	**<.0001**
1stable diatonic musician left -(2 unstable diatonic non-musician left)	1.52	0.29	108	5.19	**<.0001**
1stable diatonic musician left -(3 chromatic non-musician left)	1.53	0.29	108	5.23	**<.0001**
1stable diatonic musician left -1 stable diatonic musician right	0.96	0.29	108	3.23	**0.0073**
1stable diatonic musician left -2 unstable diatonic musician right	1.16	0.29	108	3.96	**0.0009**
1stable diatonic musician left -3 chromatic musician right	1.15	0.29	108	3.95	**0.0009**
1stable diatonic musician left -(1 stable diatonic non-musician right)	1.36	0.29	108	4.65	**0.0001**
1stable diatonic musician left -(2 unstable diatonic non-musician right)	1.41	0.29	108	4.82	**<.0001**
1stable diatonic musician left -(3 chromatic non-musician right)	1.46	0.29	108	5.01	**<.0001**
2unstable diatonic musician left- 3 chromatic musician left	0.25	0.29	72	0.89	0.596
2unstable diatonic musician left- (1 stable diatonic non-musician left)	-0.25	0.29	108	-0.85	0.596
2unstable diatonic musician left- (2 unstable diatonic non-musician left)	-0.20	0.29	108	-0.67	0.69

2unstable diatonic musician left- (3 chromatic non-musician left)	-0.18	0.29	108	-0.63	0.697
2unstable diatonic musician left- 1 stable diatonic musician right	-0.75	0.29	108	-2.56	0.052
2 unstable diatonic musician left - 2 unstable diatonic musician right	-0.55	0.29	108	-1.9	0.192
2 unstable diatonic musician left - 3 chromatic musician right	-0.56	0.29	108	-1.91	0.19
2 unstable diatonic musician left - (1 stable diatonic non-musician right)	-0.35	0.29	108	-1.21	0.44
2 unstable diatonic musician left - (2 unstable diatonic non-musician right)	-0.30	0.29	108	-1.041	0.51
2 unstable diatonic musician left - (3 chromatic non-musician right)	-0.25	0.29	108	-0.85	0.596
3 chromatic musician left - (1 stable diatonic non-musician left)	-0.50	0.29	108	-1.72	0.24
3 chromatic musician left - (2 unstable diatonic non-musician left)	-0.45	0.29	108	-1.54	0.32
3 chromatic musician left - (3 chromatic non-musician left)	-0.44	0.29	108	-1.5	0.32
3 chromatic musician left - 1 stable diatonic musician right	-1.00	0.29	108	-3.43	**0.0052**
3 chromatic musician left - 2 unstable diatonic musician right	-0.81	0.29	108	-2.76	**0.0318**
3 chromatic musician left - 3 chromatic musician right	-0.81	0.29	108	-2.78	**0.0318**
3 chromatic musician left - (1 stable diatonic non-musician right)	-0.61	0.29	108	-2.08	0.17
3 chromatic musician left - (2 unstable diatonic non-musician right)	-0.56	0.29	108	-1.91	0.19
3 chromatic musician left - (3 chromatic non-musician right)	-0.50	0.29	108	-1.71	0.24
(1 stable diatonic non-musician left) - (2 unstable diatonic non-musician left)	0.05	0.29	72	0.185	0.91
(1 stable diatonic non-musician left) - (3 chromatic non-musician left)	0.06	0.29	72	0.22	0.91
(1 stable diatonic non-musician left) - 1 stable diatonic musician right	-0.50	0.29	108	-1.71	0.24
(1stable diatonic non-musician left) - 2 unstable diatonic musician right -	0.31	0.29	108	-1.04	0.51
(1stable diatonic non-musician left) - 3 chromatic musician right	-0.31	0.29	108	-1.06	0.51
(1stable diatonic non-musician left) - (1 stable diatonic non-musician right)	-0.10	0.29	108	-0.36	0.85
(1stable diatonic non-musician left) - (2 unstable diatonic non-musician right)	-0.06	0.29	108	-0.19	0.91
(1stable diatonic non-musician left) - (3 chromatic non-musician right)	0.00	0.29	108	0.003	0.1
(2unstable diatonic non-musician left) - (3 chromatic non-musician left)	0.01	0.29	72	0.038	0.1
(2unstable diatonic non-musician left) - 1 stable diatonic musician right	-0.55	0.29	108	-1.89	0.19
(2unstable diatonic non-musician left) - 2 unstable diatonic musician right	-0.36	0.29	108	-1.23	0.44

(2unstable diatonic non-musician left) - 3 chromatic musician right	-0.36	0.29	108	-1.243	0.44
(2unstable diatonic non-musician left) - (1 stable diatonic non-musician right)	-0.16	0.29	108	-0.54	0.75
(2unstable diatonic non-musician left) - (2 unstable diatonic non-musician right)	-0.11	0.29	108	-0.371	0.8507
(2unstable diatonic non-musician left) - (3 chromatic non-musician right)	-0.05	0.29	108	-0.18	0.91
(3chromatic non-musician left) - 1 stable diatonic musician right	-0.56	0.29	108	-1.93	0.19
(3chromatic non-musician left) - 2 unstable diatonic musician right	-0.37	0.29	108	-1.27	0.45
(3chromatic non-musician left) - 3 chromatic musician right	-0.37	0.29	108	-1.28	0.45
(3chromatic non-musician left) - (1 stable diatonic non-musician right)	-0.17	0.29	108	-0.58	0.73
(3 chromatic non-musician left) - (2 unstable diatonic non-musician right)-0.119407	-0.12	0.29	108	-0.41	0.85
(3 chromatic non-musician left) - (3 chromatic non-musician right)-0.063514	-0.06	0.29	108	-0.22	0.91
1 stable diatonic musician right - 2 unstable diatonic musician right	0.19	0.29	72	0.68	0.69
1 stable diatonic musician right - 3 chromatic musician right	0.19	0.29	72	0.66	0.69
1 stable diatonic musician right - (1 stable diatonic non-musician right)	0.40	0.29	108	1.35	0.41
1 stable diatonic musician right - (2 unstable diatonic non-musician right)	0.44	0.29	108	1.52	0.32
1 stable diatonic musician right - (3 chromatic non-musician right)	0.50	0.29	108	1.71	0.24
2 unstable diatonic musician right - 3 chromatic musician right	0.00	0.29	72	-0.02	0.1
2 unstable diatonic musician right - (1 stable diatonic non-musician right)	0.20	0.29	108	0.69	0.69
2 unstable diatonic musician right - (2 unstable diatonic non-musician right)	0.25	0.29	108	0.86	0.6
2 unstable diatonic musician right - (3 chromatic non-musician right)	0.31	0.29	108	1.05	0.51
3 chromatic musician right - (1 stable diatonic non-musician right)	0.21	0.29	108	0.7	0.69
3 chromatic musician right - (2 unstable diatonic non-musician right)	0.25	0.29	108	0.872	0.596
3 chromatic musician right - (3 chromatic non-musician right)	0.31	0.29	108	1.063	0.508
(1 stable diatonic non-musician right) - (2 unstable diatonic non-musician right)	0.05	0.29	72	0.17	0.91
(1 stable diatonic non-musician right) - (3 chromatic non-musician right)	0.11	0.29	72	0.37	0.85
(2 unstable diatonic non-musician right) - (3 chromatic non-musician right)	0.06	0.29	72	0.19	0.91

Table S4: estimates, standard errors, dfs, t and p values of pairwise comparisons conducted on matchings of Experiment 4 (size explicit matchings), Tonal stability Category X Musical Experience X Circles' presentation direction. P value adjustment: Benjamini Hochberg method for 66 tests.

comparison	estimate	SE	df	t ratio	p value
1left - 2 left	0.60	0.19	34.90	3.21	**0.0424**
1left - 3 left	0.54	0.19	34.90	2.89	**0.0448**
1left - 1 right	0.13	0.19	25.60	0.70	0.61
1left - 2 right	0.25	0.17	40.40	1.45	0.26
1left - 3 right	0.36	0.17	40.40	2.08	0.13
2left - 3 left	-0.06	0.19	34.90	-0.33	0.75
2left - 1 right	-0.47	0.17	40.40	-2.75	0.04
2left - 2 right	-0.36	0.19	25.60	-1.89	0.18
2left - 3 right	-0.25	0.17	40.40	-1.44	0.26
3left - 1 right	-0.41	0.17	40.40	-2.39	0.08
3left - 2 right	-0.29	0.17	40.40	-1.71	0.20
3left - 3 right	-0.19	0.19	25.60	-0.98	0.46
1right - 2 right	0.12	0.19	34.90	0.62	0.61
1right - 3 right	0.22	0.19	34.90	1.19	0.36
2right - 3 right	0.11	0.19	34.90	0.58	0.61

Table S5: estimates, standard errors, dfs, t and p values of pairwise comparisons conducted on matchings of Experiment 4a (explicit size, musicians follow-up), Tonal stability Category X Circles' presentation direction. P value adjustment: Benjamini Hochberg method for 15 tests.

1.4. Exp. 5

comparison	estimate	SE	df	t ratio	p value
musician auditory - (non-musician auditory)	0.07	0.05	19.90	1.44	0.50
musician auditory - musician visual	0.02	0.03	15.00	0.68	0.66
musician auditory - (non-musician visual)	-0.01	0.05	19.90	-0.23	0.82
(non-musician auditory) - musician visual	-0.05	0.05	19.90	-1.07	0.60
(non-musician auditory) - (non-musician visual)	-0.08	0.03	15.00	-3.21	**0.0353**
musician visual - (non-musician visual)	-0.03	0.05	19.90	-0.60	0.66

Table S6: estimates, standard errors, dfs, t and p values of pairwise comparisons conducted on accuracy rates of Experiment 5 (size IAT), Modality X Musical Experience. P value adjustment: Benjamini Hochberg method for 6 tests.

2. Bayesian Analyses
2.1. Exp. 2 RTs

2.1.1. Bayesian Paired Samples T-Test

	BF_{10}	error %
RTs_congruent - RTs_incongruent	02.94	4.790e -8

2.1.2. Inferential Plots

Prior posterior

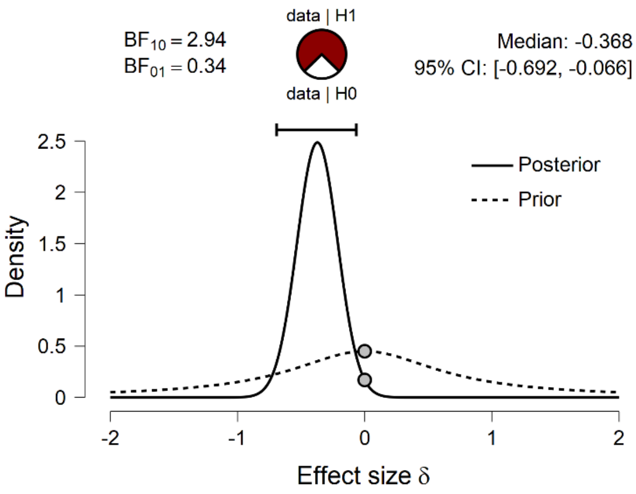

Bayes Factor Robustness Check

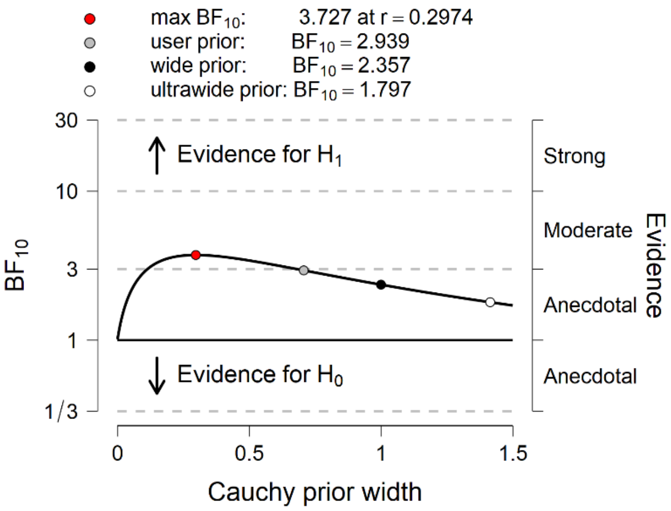

2.1.3. Descriptives

					95% Credible Interval	
	N	Mean	SD	SE	Lower	Upper
RT_con	40	609.22	78.361	12.39	584.16	634.29
RT_in	40	622.86	88.072	13.93	594.7	651.03

2.1.4. Descriptives Plots

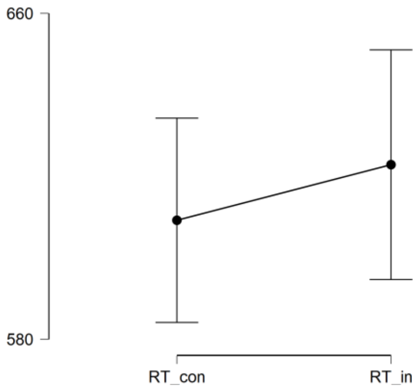

2.2. Exp. 3 Accuracy rates

2.2.1. Bayesian Paired Samples T-Test

	BF_{01}	error %
Accuracy_in - Accuracy_con	4.905	0.005

2.2.2. Inferential Plots

Prior posterior

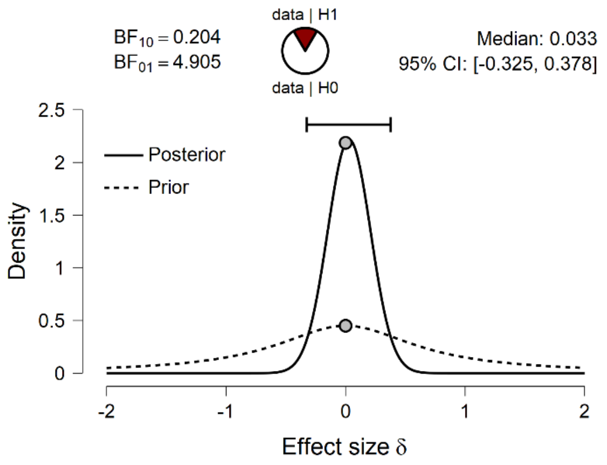

Bayes Factor Robustness Check

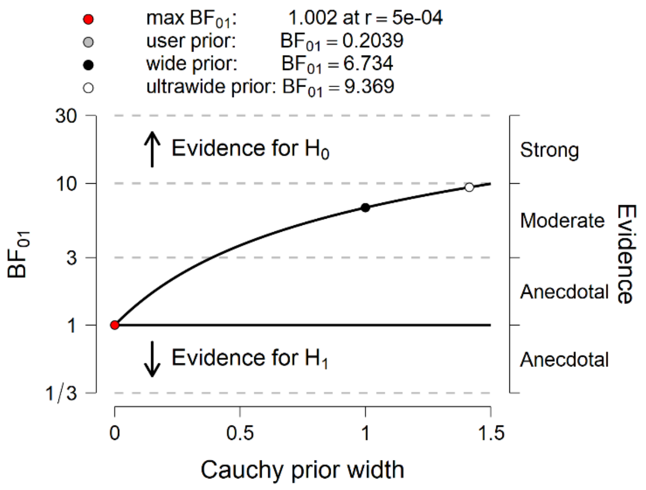

2.2.3. Descriptives

					95% Credible Interval	
	N	Mean	SD	SE	Lower	Upper
Accuracy_in	28	0.940	0.067	0.013	0.91	0.97
Accuracy_con	28	0.94	0.062	0.012	0.92	0.96

2.2.4. Descriptives Plots

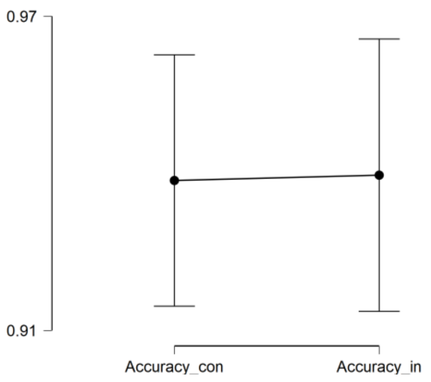

3.1. Exp. 5 RTs & Accuracy rates

3.1.2 Bayesian Paired Samples T-Test

			BF_{01}	error %
con_RT	-	in_RT	3.451	0.005
con_acc	-	in_acc	1.034	0.006

3.1.1. Inferential Plots

3.1.1.1 RTs

Prior posterior

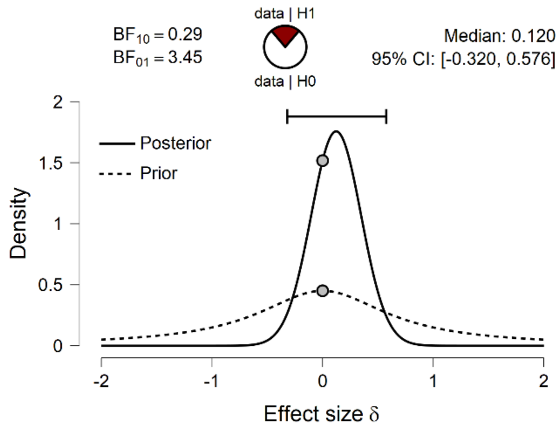

Bayes Factor Robustness Check

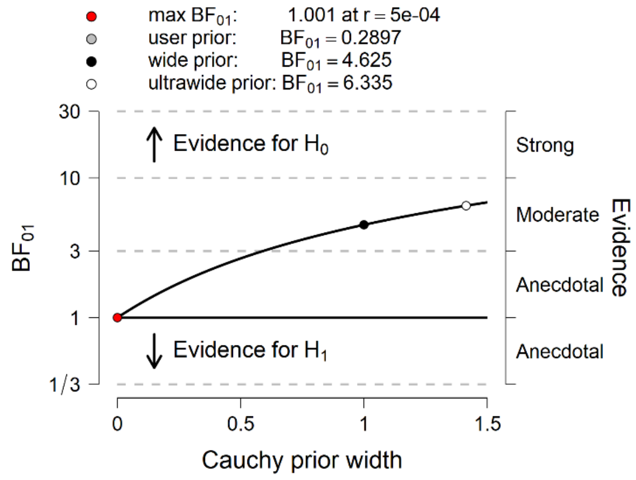

3.1.1.2. Accuracy rates

Prior posterior

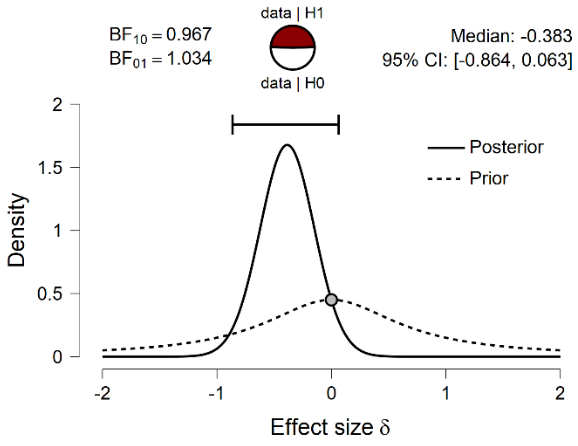

Bayes Factor Robustness Check

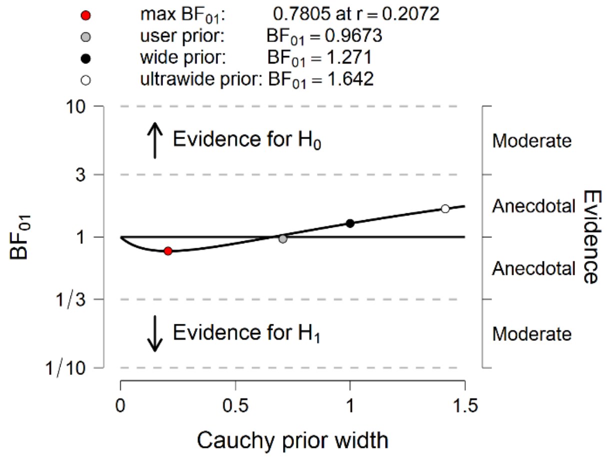

3.1.2 Descriptives

					95% Credible Interval	
	**N**	**Mean**	**SD**	**SE**	**Lower**	**Upper**
con_RT	17	523.23	58.96	14.3	492.91	553.54
in_RT	17	519.44	51.26	12.43	493.09	545.8
con_acc	17	0.87	0.10	0.03	0.81	0.92
in_acc	17	0.89	0.09	0.02	0.84	0.93

3.1.3 Descriptives Plots

RTs

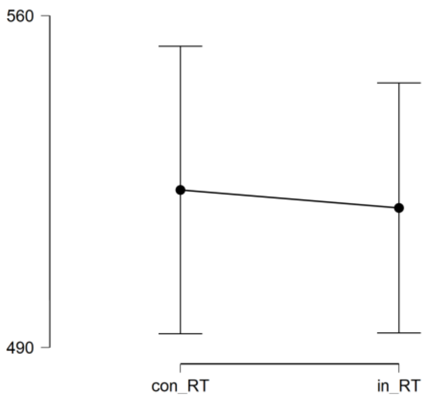

Accuracy rates

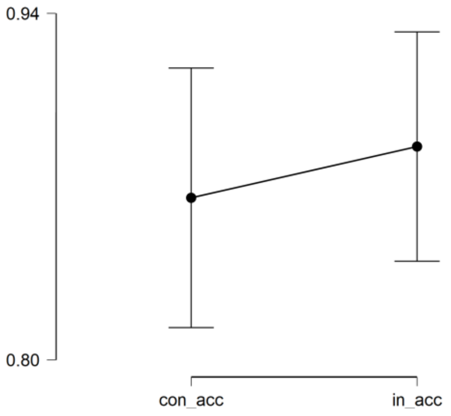

4. Sample size simulations output plots

4.1. Explicit

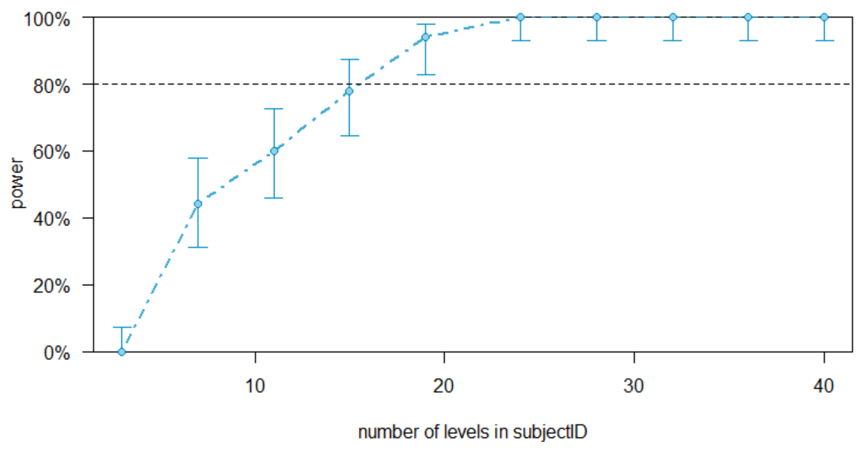

Figure S1: Sample size simulation output using simr”-- a power analysis package for r, designed to interoperate with the lme4 package for LMMs [1] based on Exp. 1 (brightness session), Maimon et al., [2].

4.2. IAT: RTs

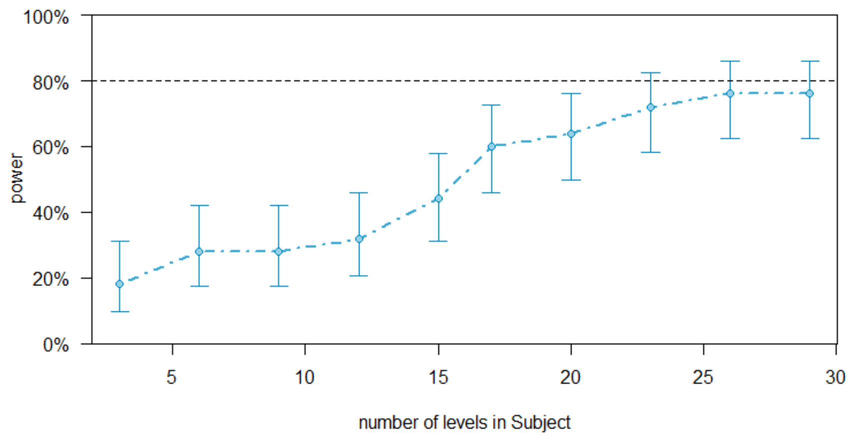

Figure S2: Sample size simulation output using simr”-- a power analysis package for r, designed to interoperate with the lme4 package for LMMs [1] based on reaction times of Exp. 2 (brightness IAT), Maimon et al., [2].

4.3. IAT: accuracy

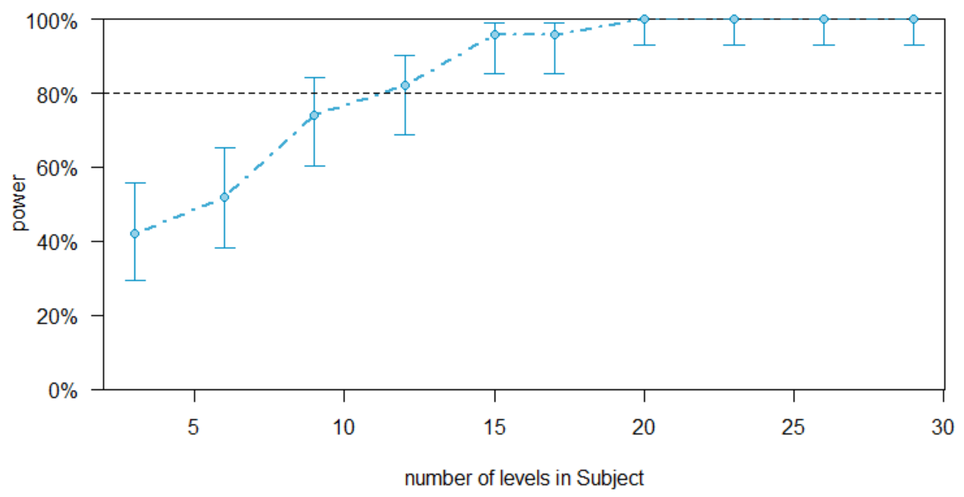

Figure S3: Sample size simulation output using simr"-- a power analysis package for r, designed to interoperate with the lme4 package for LMMs [1] based on accuracy rates of Exp. 2 (brightness IAT), Maimon et al., [2].

5. Model fit selection

	Dependent variable	slope	npar	AIC	BIC	logLik	deviance	Chi square	Df	p value
1	Y	(1 | Participant)	14	70753	70851	-35363	70725			
		(Tonal Stability | Participant)	19	70757	70890	-35360	70719	6.00	5	0.31
		(Tonal Stability + Mode | Participant)	23	70739	70900	-35347	70693	31.85	9	0.00
	X	(1 | Participant)	14	70008	70105	-34990	69980			
		(Tonal Stability | Participant)	19	69967	70099	-34964	69929	51.14	5	<0.001
		(Tonal Stability + Mode | Participant)	Failed to converge							
2	RTs	(1 | Participant)	10	275450	275530	-137715	275430			
		(Congruence | Participant)	12	275398	275494	-137687	275374	55.98	2	<0.001
		(Congruence + Modality | Participant)	15	274255	274374	-137113	274225	1149.20	3	<0.001
	Accuracy	(1 | Participant)	9	13494	13567	-6738.1	13476			
		(Congruence | Participant)	11	13490	13579	-6734.2	13468	7.85	2	0.02
		(Congruence + Modality | Participant)	14	13076	13189	-6524.1	13048	420.24	3	<0.001

3	RTs	(1 | Participant)	10	189324	189400	-94652	189304			
		(Congruence | Participant)	12	189308	189399	-94642	189284	20.23	2	<0.001
		(Congruence + Modality | Participant)	15	188161	188275	-94065	188131	1152.80	3	<0.001
	Accuracy	(1 | Participant)	9	5739.8	5808.7	-2860.9	5721.8			
		(Congruence | Participant)	11	5736.5	5820.7	-2857.3	5714.5	7.26	2	0.03
		(Congruence + Modality | Participant)	Failed to converge							
4	size	(1 | Participant)	26	30248	30428	-15098	30196			
		(Tonal Stability | Participant)	31	29868	30084	-14903	29806	389.43	5	<0.001
		(Tonal Stability+ovalDirection|Participant)	Failed to converge							
		(Tonal Stability+ovalDirection+maj_min|Participant)	Failed to converge							
4a	size	(1 | Participant)	14	16620	16709	-8295.9	16592			
		(Tonal Stability | Participant)	19	16610	16731	-8286	16572	19.94	5	0.00
		(Tonal Stability + ovalDirection|Participant)	Failed to converge							
		(Tonal Stability + ovalDirection+maj_min|Participant)	Failed to converge							
5	RTs	(1 | Participant)	10	123178	123250	-61579	123158			
		(Congruence | Participant)	12	123120	123206	-61548	123096	61.83	2	<0.001
		(Congruence + Modality | Participant)	15	123003	123111	-61486	122973	122.92	3	<0.001
	Accuracy	(1 | Participant)	9	7706.6	7772.6	-3844.3	7688.6			
		(Congruence | Participant)	11	7697	7777.7	-3837.5	7675	13.58	2	<0.001
		(Congruence + Modality | Participant)	14	7667.9	7770.6	-3819.9	7639.9	35.12	3	<0.001

Table s7: slope, number of parameters, AIC, BIC, log likelihood, deviance, Chi square, Degrees of freedom and p value of comparisons between different slopes of all mixed linear models conducted in the present study. Models which failed to converge were not inserted into the comparisons.

1. N. Maimon, D. Lamy, Z. Eitan, Crossmodal Correspondence Between Tonal Hierarchy and Visual Brightness: Associating Syntactic Structure and Perceptual Dimensions Across Modalities, *Multisensory Research*, ***33***(8), 805-836 (2020).
2. Douglas Bates, Martin Maechler, Ben Bolker, Steve Walker (2015). Fitting Linear Mixed-Effects Models Using lme4. Journal of Statistical Software, 67(1), 1-48. doi:10.18637/jss.v067.i01.
